# Piezoceramics Boosting Photocatalytic Hydrogen Evolution of Covalent Organic Frameworks

**DOI:** 10.1002/advs.202508062

**Published:** 2025-07-11

**Authors:** Jinglin Gao, Jianyu Xiao, Shijia Luo, Xuzheng Ji, Congcong Yin, Yuping Wu, Xin Zhao, Yong Wang

**Affiliations:** ^1^ School of Energy and Environment Southeast University Nanjing Jiangsu 211189 P. R. China

**Keywords:** covalent organic frameworks, photocatalytic hydrogen evolution, piezoelectricity, Z‐scheme heterojunction

## Abstract

Precise manipulation of charge‐carrier transport dynamics is a pivotal yet challenging attribute in enhancing the efficiency of energy‐conversion systems. Herein, piezopotential and optimized energy band alignment are leveraged to construct a core–shell *Z*‐scheme heterostructure, covalently bonding BaTiO_3_ nanowires (a piezoelectric ceramic) with TpPa (an imine‐linked covalent organic framework). This synergistic combination effectively overcomes the intrinsic limitations of the individual components, particularly in driving photocatalytic water splitting. The resultant *Z*‐scheme heterostructure exhibits a broadened visible‐light absorption range, finely tuned energy band alignment, abundant exposure of active sites, and enhanced piezo‐driven charge separation, collectively leading to remarkable improvements in charge‐carrier transfer and utilization efficiency. As a result, an impressive H_2_ evolution rate of 33 mmol g^−1^ h^−1^ and an outstanding apparent quantum yield of 9.39% are achieved, representing enhancements of 4.56‐fold and 10.06‐fold, respectively, compared to pure photocatalysis. This work presents an effective strategy for designing high‐efficiency catalysts and highlights the potential of piezoelectricity in boosting photo‐redox reactions.

## Graphical abstract

This study develops a core‐shell Z‐scheme piezo‐photocatalyst using covalent interfacial bridging between piezoelectric ceramics and covalent organic frameworks. Proper band alignment enables efficient electron transfer and internal electric field formation, while piezoelectricity generates a polarization field under ultrasound, enhancing solar energy utilization and catalytic activity synergistically.

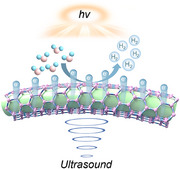

## 1 Introduction

Precise manipulation of charge‐carrier transport dynamics serves as a critical design paradigm for optimizing quantum efficiency in electrochemically driven photocatalytic systems. Such control modality is typically achieved through the real‐time adjustment of band bending via programmable bias potentials and/or the establishment of unidirectional charge‐transfer pathways at heterojunction interfaces.^[^ ^1^ ^]^ Despite notable advancements in inorganic conductor‐based photocatalysts such as metal sulfides,^[^ ^2^ ^]^ metal oxides,^[^ ^3^ ^]^ and their composites,^[^ ^4^ ^]^ the majority of these materials still exhibit limited visible‐light absorption, inefficient charge‐carrier utilization, and inadequate exposure of active sites. Therefore, the development of high‐efficiency photocatalysts with rationally optimized energy band structures and sustained driving forces remains essential for achieving superior redox performance.

Covalent organic frameworks (COFs) are porous crystalline materials precisely assembled from periodic skeletons interconnected via covalent bonds.^[^ ^5^ ^]^ Their strong conjugation and abundant functional molecular units endow COFs with exceptional light‐harvesting capabilities and tunable energy band structures,^[^ ^6^ ^]^ making them a highly promising platform for photocatalytic and optical applications.^[^ ^7^ ^]^ Recently, COFs have attracted considerable attention in the realm of photo‐redox catalysis, especially for applications like water splitting and CO_2_ reduction, where they have demonstrated remarkably high activity compared to their inorganic counterparts.^[^ ^8^ ^]^ Despite these advancements, the widespread application of COF‐based materials in photo‐redox reactions remains hindered by challenges such as limited carrier transfer pathways and rapid recombination between electrons and holes.^[^ ^9^ ^]^ To address these dilemmas, the construction of rational heterostructures—such as COFs/metal sulfides,^[^ ^10^ ^]^ COFs/metal oxides,^[^ ^11^ ^]^ and COFs/COFs^[^ ^12^ ^]^—has emerged as an effective strategy to overcome the inherent drawbacks of pure COF photocatalysts. Although these approaches optimize carrier migration pathways and achieve adsorption equilibrium of reaction intermediates, the overall solar energy utilization efficiency is still constrained by insufficient carrier transfer dynamics.^[^ ^13^ ^]^ Hence, it is imperative to incorporate an additional driving force to further accelerate electron transfer to the catalytic surface, thereby improving the overall performance of COF‐based systems.

Internal electric fields (IEFs), arising from asymmetric charge redistribution, play a pivotal role in facilitate various photo(electro)catalytic processes. These fields offer significant advantages, including overcoming reaction kinetic barriers, accelerating carrier transfer dynamics, and reducing redox potentials.^[^ ^14^ ^]^ Among these IEFs, piezoelectric polarization can be initiated by periodic mechanical stress. This process can induce band bending, initiate the release of bound charges, and lead to the formation of the polarization fields. In the IEFs, the direction of electron transfer is opposite to that of the generated field.^[^ ^15^ ^]^ Recently, the piezoelectric effect has been widely explored in emerging organic pollutants degradation,^[^ ^16^ ^]^ advanced oxidation process,^[^ ^17^ ^]^ energy harvesting and transformation.^[^ ^18^ ^]^ However, despite the presence of a strong driving force, achieving a desirable catalytic rate remains challenging, particularly for small‐molecule transformations. This limitation arises from constrained adsorption dynamics and suboptimal energy band structures.^[^ ^19^ ^]^

The integration of piezoelectric effects with photocatalysis has emerged as a highly promising approach to overcoming these challenges.^[^ ^20^ ^]^ To date, the majority of piezo‐photocatalysts in the literature is mainly composed of inorganic materials.^[^ ^21^ ^]^ While these materials have demonstrated improved carrier transfer efficiency, their redox performance is often limited by insufficient active adsorption sites and poor visible‐light harvesting capabilities. In this context, COFs, with the large surface areas and tunable energy band structures, present an intriguing opportunity. By combining COFs with piezoelectric materials, it is anticipated that a synergistic effect can be achieved, significantly boosting redox reactions and addressing the current limitations of piezo‐photocatalytic systems.

In this study, we strategically engineered a core–shell *Z*‐scheme piezo‐photocatalyst via covalent interfacial bridging between BaTiO_3_ nanowires (a piezoelectric ceramic) with TpPa (an imine‐linked covalent organic framework). This unique architecture synergistically combines the piezoelectric properties of BaTiO_3_ with the photo‐responsive advantages of TpPa, achieving a remarkable enhancement in hydrogen evolution rates (33 mmol g^−1^ h^−1^) and apparent quantum yield (9.39%), exceeding the activity of recently reported state‐of‐the‐art piezo‐photocatalysts.

## 2 Results and Discussion

### 2.1 Synthesis and Characterization of TpPa‐Encapsulated BaTiO_3_ Nanowires

**Figure 1:**
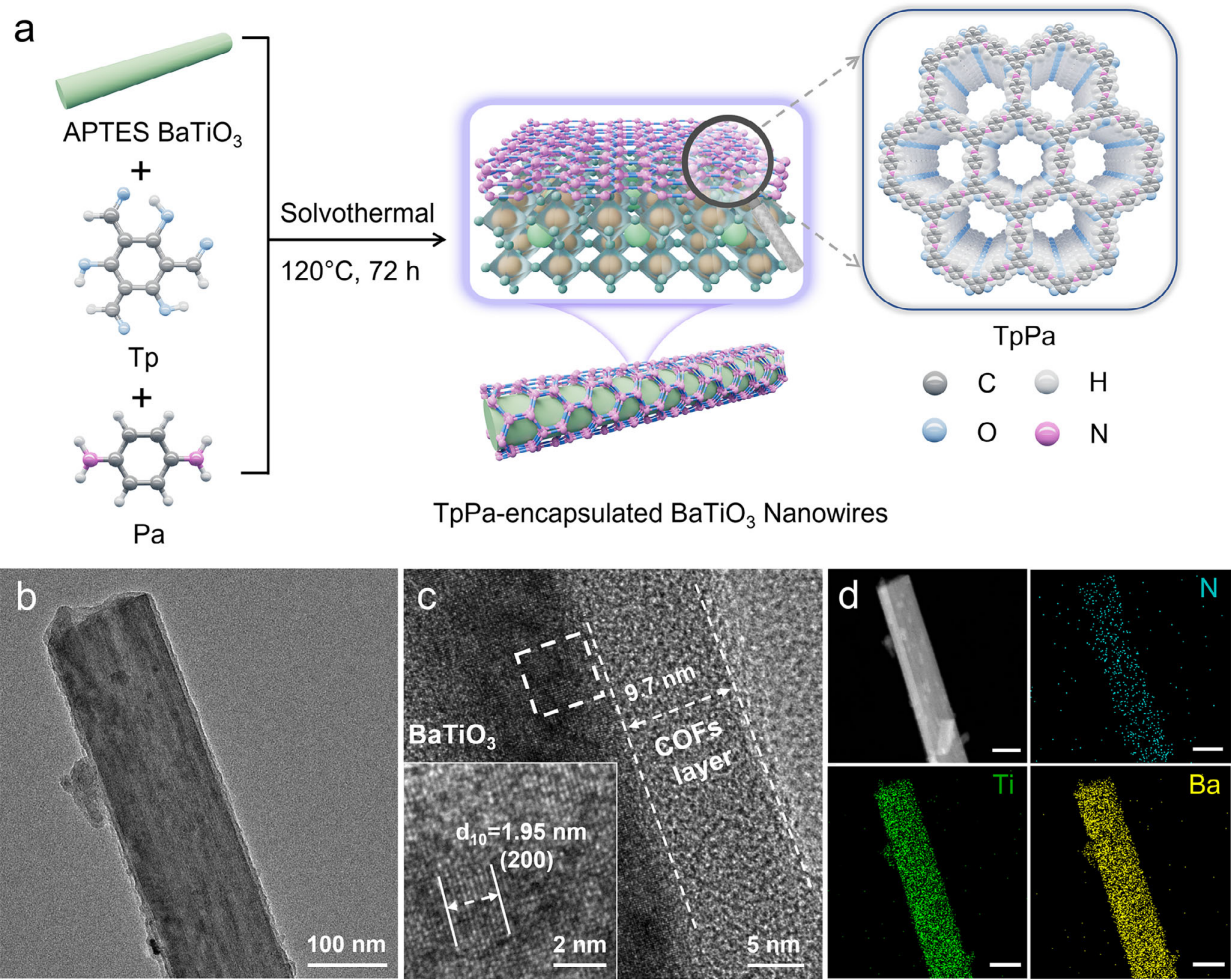
Synthesis and characterization of TpPa‐encapsulated BaTiO_3_ nanowires. a) Schematic diagram of the synthesis process for TpPa‐encapsulated BaTiO_3_ nanowires. b) TEM and c) HRTEM images of BTW‐40@TpPa. d) High‐angle annular dark field image and the corresponding EDS mapping of BTW‐40@TpPa. The scale bars are 100 nm.

The schematic illustration of the synthetic process for TpPa‐encapsulated BaTiO_3_ nanowires is presented in **Figure** **1**a. First, the BaTiO_3_ nanowires were synthesized using a reported two‐step hydrothermal method, with TiO_2_ and Ba(OH)_2_·8H_2_O as precursors.^[^ ^22^ ^]^ The scanning electron microscopy (SEM) is used to observe the morphology of BaTiO_3_ nanowires. Figure S1 (Supporting Information) reveals that BaTiO_3_ displays well‐defined nanowires with micrometer‐scale lengths (>1 µm) and nanometer‐scale diameters (40–110 nm). These nanowires, possessing a high aspect ratio, exhibit enhanced stress responsiveness compared to nanocubes or nanospheres, enabling the generation of a stronger piezopotential under mechanical stress.^[^ ^23^ ^]^ Finite element analysis further confirmed the superior piezoresponse of nanowires when subjected to an identical boundary loading of 10^8^ N m^−2^ (Figure S2, Supporting Information). Notably, the maximum surface potential of the nanowires exceeds that of other morphologies with similar dimensions by 5–10 times. The successful preparation of BaTiO_3_ nanowires was further validated through powder X‐ray diffraction (PXRD) and high‐resolution transmission electron microscopy (HRTEM). All diffraction peaks in Figure S3 (Supporting Information) are unambiguously assigned to the tetragonal crystal structure of BaTiO_3_ (JCPDS No. 05–0626). Additionally, HRTEM analysis reveals an interplanar spacing of 0.39 nm, corresponding to the lattice fringes of the (100) plane of BaTiO_3_, thereby providing direct evidence for the material's crystallinity and phase purity. The PXRD patterns also revealed that TpPa exhibits a high degree of crystallinity (Figure S4a, Supporting Information). Characteristic diffraction peaks were observed at 4.6°, 8.1°, and 26.1°, which can be indexed to the (100), (110), and (001) crystallographic facets, respectively. A comparison of the experimental PXRD profile of TpPa with simulated profiles corresponding to different stacking models (Figure S4b,c, Supporting Information) indicated that the experimental data are best matched by the eclipsed AA stacking mode. Full‐profile Pawley refinement performed in the hexagonal P6/m space group yielded unit cell parameters of *a* = *b* = 22.04 Å and *c* = 3.49 Å. The refinement showed excellent agreement with the experimental data, as evidenced by the low R‐factors: weighted profile R‐factor (*R*_wp_) = 5.50% and unweighted R‐factor (*R*_p_) = 3.78% for TpPa.

Prior to the encapsulation of BaTiO_3_ nanowires with TpPa, the nanowires were surface‐functionalized with 3‐aminopropyltriethoxysilane (APTES) to introduce abundant amine groups.^[^ ^24^ ^]^ Subsequently, the APTES‐BaTiO_3_ was incorporated into the reported solvothermal synthetic system of TpPa to obtain final covalently bonded core–shell structure via Schiff base reaction.^[^ ^25^ ^]^ To verify the covalent linkage between BaTiO_3_ and TpPa, UV–vis diffuse reflectance spectroscopy (DRS) was performed on a physical mixture of TpPa and BaTiO_3_ as a control sample. As shown in Figure S5 (Supporting Information), the physical mixture, without any covalent bonding between the two components, exhibits two distinct reflectance edges corresponding to BaTiO_3_ and TpPa individually. In contrast, BTW‐40@TpPa displays an enhanced absorption band with a single, well‐defined reflectance edge that closely resembles that of pristine TpPa. This observation strongly suggests that the integration of BaTiO_3_ and TpPa occurs through covalent bonding rather than simple physical mixing.^[^ ^26^ ^]^ To further investigate the formation of covalent bonds and the associated changes in chemical bonds, we synthesized a Tp‐BaTiO_3_ hybrid material using Tp and APTES‐functionalized BaTiO_3_ via a solvothermal method. The resulting Tp‐BaTiO_3_ hybrid exhibited a notable disappearance of the characteristic absorption peaks corresponding to the N─H stretching vibration (from APTES‐ BaTiO_3_) and C═O groups (from TpPa), along with the emergence of new absorption bands attributable to C═N and C─N bonds (Figure S6, Supporting Information). These results provide additional evidence that Tp has been successfully grafted onto the surface of BaTiO_3_ through covalent bonding. Such robust covalent linkages provide enhanced stability during the piezo‐photocatalytic process.

The SEM and TEM images of BTW‐X@TpPa (X = 20, 40, and 60 represents the relative mass of BaTiO_3_ nanowires in the core–shell structure) clearly reveal that the surface of BaTiO_3_ nanowires is uniformly encapsulated with TpPa (Figure 1b; Figure S7, Supporting Information). HRTEM analysis further demonstrates that the thickness of the TpPa layer is predominantly determined by the mass ratio of BaTiO_3_ nanowires to TpPa, exhibiting significant variation in the range of 5.4–11.6 nm (Figure 1c; Figure S8, Supporting Information). The shell thickness in BTW‐40@TpPa was measured to be ≈9.7 nm. The distinct lattice fringes corresponding to the (200) crystallographic plane of BaTiO_3_ adjacent to the TpPa layer provide direct evidence for the successful encapsulation of BaTiO_3_ nanowires by the COF material. Additionally, energy dispersive spectrometer (EDS) mapping analysis reveals the uniform distribution of N (from TpPa), Ti, and Ba (from BaTiO_3_), further validating the complete encapsulation of the nanowires by the COF layer and the formation of well‐defined core–shell structures (Figure 1d). These structural characteristics, coupled with the observed core–shell morphology, conclusively demonstrate the successful fabrication of heterojunctions with an intact interfacial contact, highlighting the precision and effectiveness of the synthetic approach.

Fourier transform infrared (FT‐IR) spectroscopy and X‐ray photoelectron spectroscopy (XPS) offered atomic‐level insights into the chemical states and interfacial charge transfer within the core–shell structure. In the FT‐IR spectrum of TpPa (Figure S9, Supporting Information), the absorption peak observed at 1630 cm^−1^ can be attributed to C═O stretching vibrations. The C─N peak appeared at 1253 cm^−1^, while the N─H stretching signals at 3374, 3304, and 3201 cm^−1^ were absent, indicating the successful Schiff base reaction. Additionally, the absorption bands in the range of 400–500 cm^−1^ in BaTiO_3_ correspond to the stretching vibration modes of the Ti─O bond. Notably, all these characteristic peaks were preserved in the heterostructures, indicating the successful integration of BaTiO_3_ and TpPa. The PXRD patterns further corroborate the formation of the heterostructures, as evidenced by the presence of diffraction peaks associated with both components (Figure S10, Supporting Information). The XPS survey spectrum of BTW‐40@TpPa reveals the presence of C, O, N, Ba, and Ti without any detectable impurity peaks (Figure S11, Supporting Information). Analysis of the high‐resolution XPS spectra for each element provides deeper insights into the interfacial interactions. Specifically, the binding energies of C 1s, N 1s, and O 1s in BTW‐40@TpPa exhibit a more negative shift compared to those in TpPa, while the binding energies of Ba 3d and Ti 2p show a positive shift. These variations in binding energy can be attributed to electrostatic coupling effects, indicating electron transfer from BaTiO_3_ to TpPa. The interfacial charge redistribution establishes a permanent internal electric field (IEF) across the heterojunction,^[^ ^27^ ^]^ which is of great significance in promoting charge separation and boosting catalytic performance.

Moreover, thermogravimetric analysis (TGA) under a nitrogen atmosphere was conducted to determine the mass ratio of BaTiO_3_ and TpPa in the heterostructures (Figure S12, Supporting Information). For BTW‐40@TpPa, a mass loss of 55% was observed at a heat‐treatment temperature of 800 °C, indicating the complete decomposition of TpPa. The remaining mass, attributed to BaTiO_3_, was ≈45%, which closely aligns with the theoretical value of 40%. To evaluate the exposure of active adsorption sites for the H_2_ evolution reaction, N_2_ adsorption isotherms were measured at 77 K (Figure S13, Supporting Information). The Brunauer–Emmett–Teller (BET) surface area of pure TpPa was determined to be 721.2 m^2^ g^−1^ by NLDFT model analysis, while the heterostructures showed a slight reduction in surface area due to the incorporation of BaTiO_3_.^[^ ^28^ ^]^ Notably, the synthesized BTW‐40@TpPa maintained a considerable BET surface area of 534.7 m^2^ g^−1^, thereby offering abundant exposure of active sites to effectively activate water molecules during hydrogen evolution.

**Figure 2:**
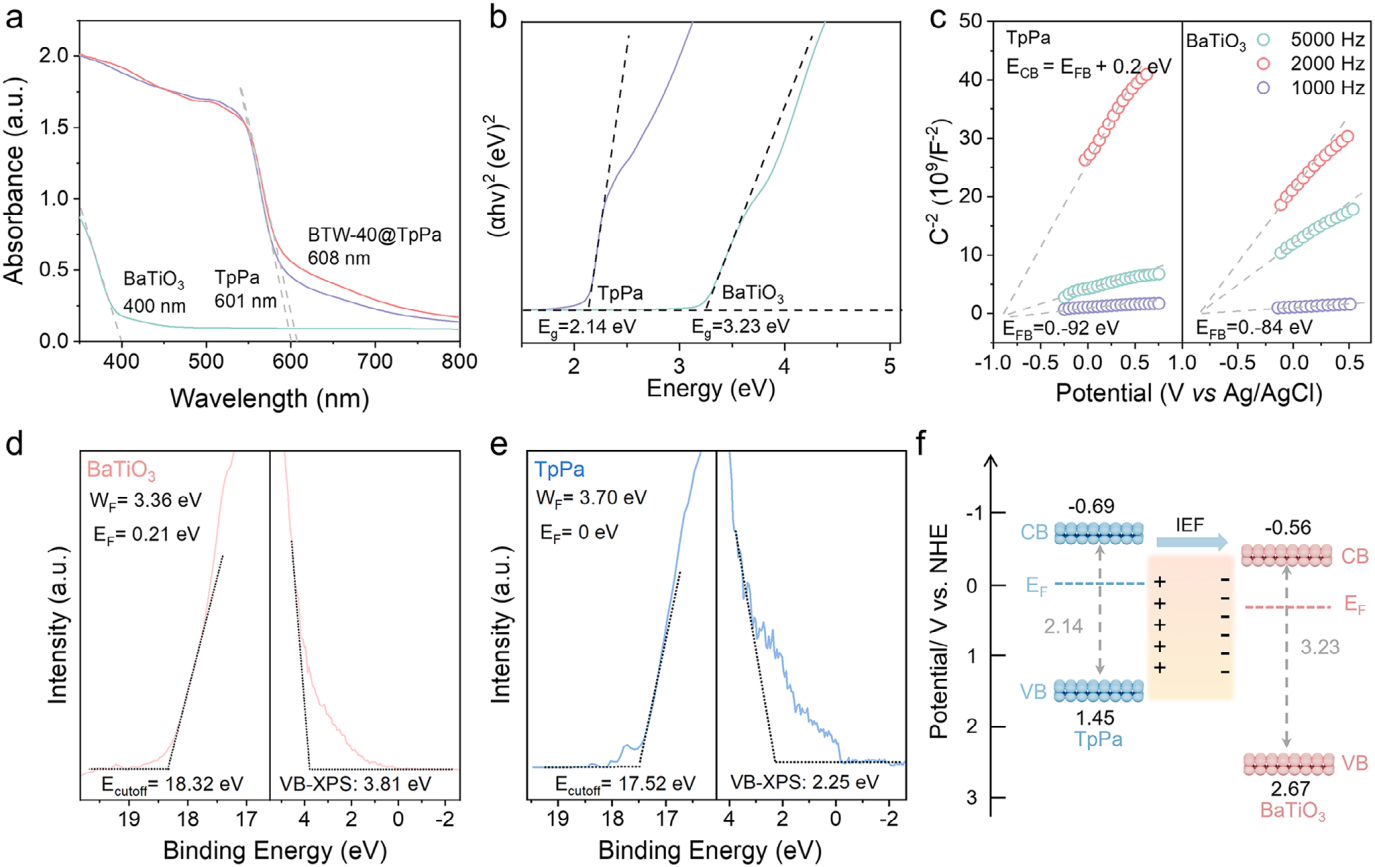
Band structures of BaTiO_3_, TpPa, and BTW‐40@TpPa. a) UV−vis diffuse reflectance absorption spectra of BTW‐40@TpPa, BaTiO_3_, and TpPa. b) Tauc plots of TpPa and BaTiO_3_. c) Mott−Schottky plots of TpPa and BaTiO_3_. UPS and VB‐XPS spectra of d) BaTiO_3_ and e) TpPa. f) The corresponding energy band structure.

The light‐harvesting capacities of BaTiO_3_, TpPa, and BTW‐40@TpPa were further evaluated using UV–vis DRS. As depicted in **Figure** **2**a, BaTiO_3_ exhibits a minimal visible‐light absorption capacity, while BTW‐40@TpPa demonstrates a notable red‐shift, with an optical absorption edge occurring at ≈608 nm—extending beyond that of pure TpPa. This spectral shift indicates that the visible‐light harvesting capability of BaTiO_3_ is enhanced within the heterojunction structure.^[^ ^29^ ^]^ The bandgap energies of pristine TpPa and BaTiO_3_ nanowires were determined to be 2.14 and 3.23 eV, respectively, using the Kubelka–Munk equation (Figure 2b).^[^ ^30^ ^]^ Furthermore, the conduction band (CB) potentials of TpPa and BaTiO_3_ were estimated to be −0.72 and −0.64 eV, respectively, through Mott‐Schottky analysis (Figure 2c). This complementary band alignment between TpPa and BaTiO_3_ facilitates to generate a *Z*‐scheme electron transfer pathways accompanied by an IEF.^[^ ^31^ ^]^ To further determine the energy band alignment between BaTiO_3_ and TpPa, ultraviolet photoelectron spectroscopy (UPS) and valence band (VB) XPS were carried out in combination with DRS results. As shown in Figure 2d,e, the work functions (W_F_) of BaTiO_3_ and TpPa were determined to be 3.36 and 3.70 eV, respectively. The VB maxima position relative to the Fermi level (E_F_) were found to be 3.81 eV for BaTiO_3_ and 2.25 eV for TpPa. Accordingly, the VB edge potentials of BaTiO_3_ and TpPa were calculated to be 2.67 and 1.45 eV versus NHE, respectively, which are consistent with the values obtained from electrochemical measurements. Based on the DRS results, the CB potentials of BaTiO_3_ and TpPa were estimated to be −0.56 and −0.69 eV versus NHE, respectively. This aligns well with the CB position inferred from the Mott–Schottky plot. These values reveal a staggered band alignment at the interface between BaTiO_3_ and TpPa (Figure 2f). Furthermore, the differences in E_F_ drive electron transfer from BaTiO_3_, which has a higher value, to TpPa, which has a lower value. Upon reaching equilibrium, the Fermi levels align with a minimal offset, suggesting the formation of a stable Z‐scheme heterojunction and the presence of an IEF at the interface, which facilitates efficient charge separation.

### 2.2 Piezo‐Photocatalytic Activity

**Figure 3:**
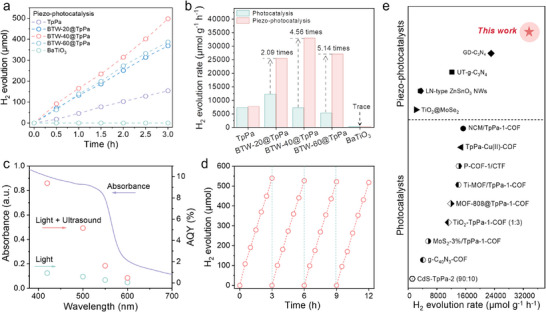
Piezo‐photocatalytic H_2_ evolution performance. a) Time‐dependent piezo‐photocatalytic H_2_ evolution for BaTiO_3_, TpPa, BTW‐20@TpPa, BTW‐40@TpPa, and BTW‐60@TpPa. b) Comparison of H_2_ evolution rate of photocatalysis and piezo‐photocatalysis. c) Apparent quantum yield of photocatalytic and piezo‐photocatalytic H_2_ production for BTW‐40@TpPa. d) Recycling tests of BTW‐40@TpPa for piezo‐photocatalytic H_2_ evolution. e) Comparison of H_2_ production performance of BTW‐40@TpPa with the reported representative photocatalysts and piezo‐photocatalysts.

The piezocatalytic, photocatalytic, and piezo‐photocatalytic performances of BaTiO_3_ nanowires, TpPa, and their heterostructures were systematically evaluated through their ability to activate water molecules for H_2_ production. The temperature of reaction system was kept at 5 °C using circulating cooling water, while periodic mechanical stress was applied via an ultrasonic cleaner (Figure S14, Supporting Information). The pure BaTiO_3_ nanowires failed to achieve photocatalytic H_2_ evolution because the pure BaTiO_3_ cannot be excited to generated photoelectrons under visible light irradiation (Figure S15, Supporting Information). In contrast, TpPa successfully initiated the photoreduction reaction, driven by abundant photoexcited electrons. Notably, the H_2_ production performance of the heterostructures exhibited a declining trend with increasing BaTiO_3_ nanowire content. The highest H_2_ evolution rate was observed for BTW‐20@TpPa, owing to its interfacial Z‐scheme charge transfer dynamics, which synergistically improve carrier separation efficiency. However, further increasing the mass ratio of BaTiO_3_ nanowires led to a reduction in photocatalytic activity, likely due to insufficient exposure of catalytic active sites. Under sole ultrasonic excitation (45 kHz, 300 W), all samples exhibited a negligible water‐splitting activity, as evidenced by the absence of detectable H_2_ evolution (Figure S16, Supporting Information). The underlying mechanism can be attributed to the fact that the generated piezopotential falls below the critical threshold required to drive the water splitting reaction to overcome the thermodynamic limitations.^[^ ^1b^ ^]^ Remarkably, when both light irradiation and ultrasound were applied simultaneously, the H_2_ evolution capacity of the piezo‐photocatalysts was significantly enhanced, surpassing the performance of individual piezocatalysis or photocatalysis (**Figure** **3**a,b). Among the tested samples, BTW‐40@TpPa demonstrated an impressive H_2_ evolution rate of 33 mmol g^−1^ h^−1^, which was 4.56 times higher than counterparts solely under light irradiation without ultrasound. Furthermore, a large number of H_2_ bubbles were clearly observed in the reaction system (Video S1, Supporting Information), further confirming the excellent piezo‐photocatalytic H_2_ production activity. These results highlight the synergistic effect of ultrasound and visible light in enhancing the catalytic performance of piezo‐photocatalysts beyond that of pristine piezo or photocatalysts. Simultaneously, BTW‐40@TpPa exhibited an outstanding apparent quantum yield (AQY) of 9.39% at 420 nm (Figure 3c; Tables S1 and S2, Supporting Information), which is 10.06 times higher than that achieved solely under light irradiation. This suggests that the presence of the ultrasound‐responsive piezopotential effectively facilitates the transport and separation of photogenerated carriers, thereby enhancing the utilization efficiency of electrons in the photoreduction reaction. The recycling and long‐term piezo‐photocatalytic tests (Figure 3d; Figures S17, Supporting Information) confirmed that BTW‐40@TpPa maintains excellent catalytic stability. More importantly, comprehensive characterizations, including SEM and TEM imaging, XPS analysis, PXRD patterns, and BET surface area measurements (Figures S18 and S19, Supporting Information), both before and after 20 h of reaction, reveal that BTW‐40@TpPa retains its structural integrity. To further verify the influence of the piezoelectric effect on catalytic performance, we synthesized two control heterojunctions: a piezoelectric system, BTW‐40@TpBD, and a non‐piezoelectric counterpart, TiO_2_‐40@TpPa. As shown in Figures S20a and S21a (Supporting Information), both materials exhibit a core–shell architecture with a COF layer encapsulating the respective inorganic cores. The formation of these heterostructures is corroborated by PXRD analysis, which reveals characteristic diffraction peaks corresponding to both components in each system (Figures S20b and S21b, Supporting Information). Under combined light and ultrasound irradiation, the piezoelectric BTW‐40@TpBD heterojunction achieved a notable piezo‐photocatalytic H_2_ evolution rate of 23.8 mmol g^−1^ h^−1^, representing a 2.01‐fold enhancement compared to that under light irradiation alone (Figure S20c,d, Supporting Information). In contrast, the non‐piezoelectric TiO_2_‐40@TpPa exhibited no significant improvement in catalytic activity under the same conditions (Figure S21c,d, Supporting Information). These results provide strong experimental evidence that the observed performance enhancement originates from the piezoelectric potential generated under ultrasound irradiation, thereby validating its crucial role in promoting photocatalytic H_2_ evolution.

To gain deeper insight into the role of covalent bonding in the piezo‐photocatalytic process, comparative experiments were carried out using a physical mixture of TpPa and BaTiO_3_ under identical experimental conditions. As shown in Figure S22 (Supporting Information), the physical mixture exhibits a lighter color compared to BTW‐40@TpPa, which may indicate reduced light absorption capacity. Under the same conditions, the catalytic activity of BTW‐40@TpPa was found to be significantly higher than that of the physically mixed counterpart. This enhanced performance highlights the crucial role of the strong covalent bonds in facilitating efficient charge transfer and coupling between the piezoelectric and photocatalytic components, thereby enhancing the overall piezo‐photocatalytic efficiency. Considering the influence of mechanical force frequency on alternating polarization fields,^[^ ^32^ ^]^ the impact of ultrasonic frequency (45, 80, and 100 kHz) on H_2_ evolution performance was investigated. As shown in Figure S23 (Supporting Information), the maximum H_2_ production amount was observed at 45 kHz, indicating that this frequency is closer to the characteristic resonance frequency of the material,^[^ ^33^ ^]^ thereby inducing a stronger polarization field to maximize electron‐hole separation. Ultrasonic power also plays a critical role in the piezo‐photocatalytic process. As shown in Figure S24 (Supporting Information), the catalytic activity of pure TpPa showed negligible improvement across all tested power levels, indicating that triboelectric effects have minimal influence on the reaction enhancement. In contrast, piezo‐photocatalytic experiments on BTW‐40@TpPa conducted at varying ultrasonic power levels reveal a clear increase in catalytic activity with increasing power (Figure S25, Supporting Information). Notably, all activity values under ultrasound‐assisted conditions surpass those observed under pure photocatalytic conditions, further confirming the positive contribution of the piezoelectric effect to overall catalytic efficiency. Furthermore, the comprehensive comparisons with previously reported piezo/photocatalytic materials (Figure 3e; Table S3, Supporting Information) reveals that BTW‐40@TpPa exhibits exceptional H_2_ production performance among state‐of‐the‐art piezo/photocatalysts. These findings underscore the significant potential of coupling piezoelectric materials with COFs to activate water molecules by leveraging solar and mechanical energy.

### 2.3 Photoelectrochemical and Piezoelectric Properties

**Figure 4:**
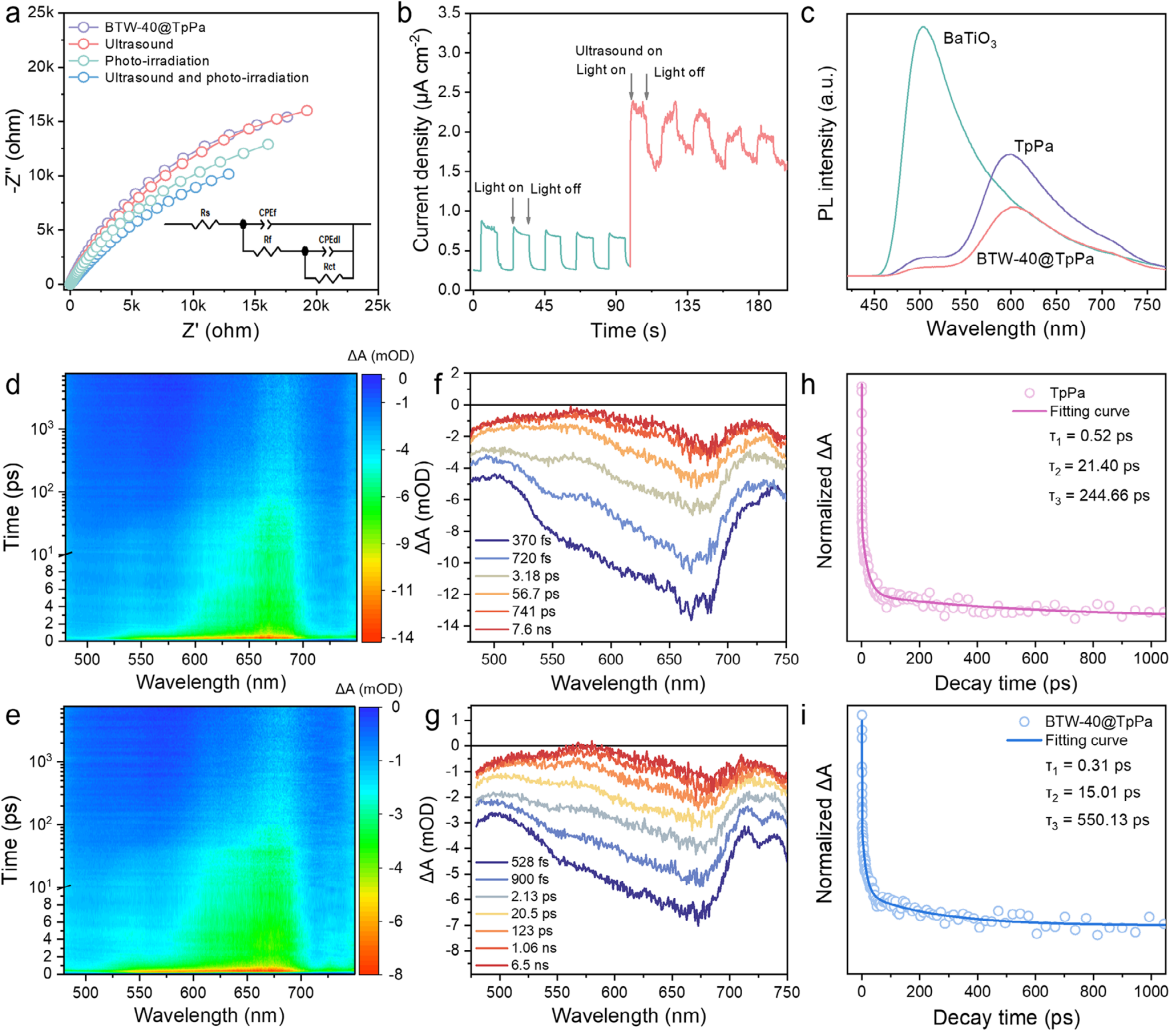
Photoelectrochemical characterizations and carriers transfer dynamics. a) Electrochemical impedance spectroscopy and b) current‐time curve of BTW‐40@TpPa under simultaneous ultrasound and light illumination. c) PL spectra of BaTiO_3_, TpPa, and BTW‐40@TpPa. 2D mapping transient absorption spectroscopy of d) TpPa, and e) BTW‐40@TpPa. Transient absorption spectra recorded at indicated delay times measured upon 400 nm excitation of f) TpPa, and g) BTW‐40@TpPa. Transient absorption traces for h) TpPa, and i) BTW‐40@TpPa normalized to the 550 nm exciton bands.

To obtain comprehensive insights into the precise role and potential enhancement mechanisms of ultrasound‐assisted photocatalysis, a series of photoelectrochemical and piezoelectric measurements were systematically conducted. Initially, electrochemical impedance spectroscopy (EIS) was performed using a three‐electrode system (**Figure** **4**a; Figure S26, Supporting Information). The distinct stimulus‐responsive characteristics of BaTiO_3_ and TpPa are evident in their impedance behaviors: BaTiO_3_, which is mechanically responsive, demonstrates a significant reduction in impedance during ultrasonic treatment, while remaining unaffected by light illumination. Conversely, the photo‐responsive TpPa exhibits a significant impedance attenuation under light exposure but demonstrates a negligible response to mechanical stimulation. In comparison with pristine BaTiO_3_ and TpPa, BTW‐40@TpPa displays smaller Nyquist plot radii that can be fitted to due to the formation of a *Z*‐scheme heterojunction, indicating a relatively lower interfacial charge transfer resistance and faster carrier transport. Further, the EIS data was fitted well to the equivalent circuit using Zview software (Table S4, Supporting Information). This resistance (*R*_ct_) is further reduced when subjected to simultaneous ultrasound and visible light illumination, revealing desirable synergistic responses to both ultrasonic and light stimuli. This synchronized response is also reflected in the current density measurements. The transient piezo‐photocurrent density of BTW‐40@TpPa significantly increases by more than threefold compared to that under single light illumination (Figure 4b; Figure S27, Supporting Information), further demonstrating the rapid transmission of photogenerated electrons facilitated by the synergistic effects of the piezopotential and IEF. Moreover, weaker composite photoluminescence (PL) signals and shorter fluorescence lifetimes further suggest that such *Z*‐scheme heterostructures effectively accelerate intramolecular charge transfer and reduce the likelihood of photoinduced excited‐state electron inactivation before they reach the reactive sites (Figure 4c; Figure S28, Supporting Information). Transient absorption spectroscopy (TAS) was employed to investigate the decay dynamics of photogenerated charge carriers and their corresponding lifetimes. A 400 nm pump pulse was used for excitation, and the results were presented in the form of 2D pseudocolor plots (Figure 4d,e), along with representative decay profiles at selected detection wavelengths (Figure 4f,g). The time‐resolved signals at a probe wavelength of 550 nm were fitted using a triple‐exponential decay model, suggesting the presence of three distinct relaxation pathways for the photogenerated electrons (Figure 4h,i). The shortest lifetime (τ_1_) was attributed to the cooling of hot electrons to the bottom of the conduction band. The intermediate lifetime (τ_2_) corresponded to fast charge recombination, while the longest lifetime (τ_3_) reflected the trapping of electrons in shallow states.^[^ ^27^ ^]^ In BTW‐40@TpPa, the hot electron cooling process (τ_1_ = 0.31 ps) was comparable to that observed in pure TpPa (τ_1_ = 0.52 ps). However, the charge recombination lifetime in BTW‐40@TpPa (τ_2_ = 15.01 ps) was significantly shorter than that in pure TpPa (τ_2_ = 21.40 ps), which can be attributed to interfacial charge recombination from the CB of BaTiO_3_ to the VB of TpPa. In contrast, the lifetime associated with shallow electron trapping in BTW‐40@TpPa was notably extended (τ_3_ = 550.13 ps) compared to TpPa (τ_3_ = 244.66 ps), indicating a more stable retention of photogenerated electrons. This prolonged trapping state allows a greater number of electrons to participate in photocatalytic reduction reactions. As a result, the enhanced charge separation efficiency in BTW‐40@TpPa leads to improved photocatalytic performance, highlighting the beneficial role of the heterojunction structure in promoting electron utilization during photochemical processes.

**Figure 5:**
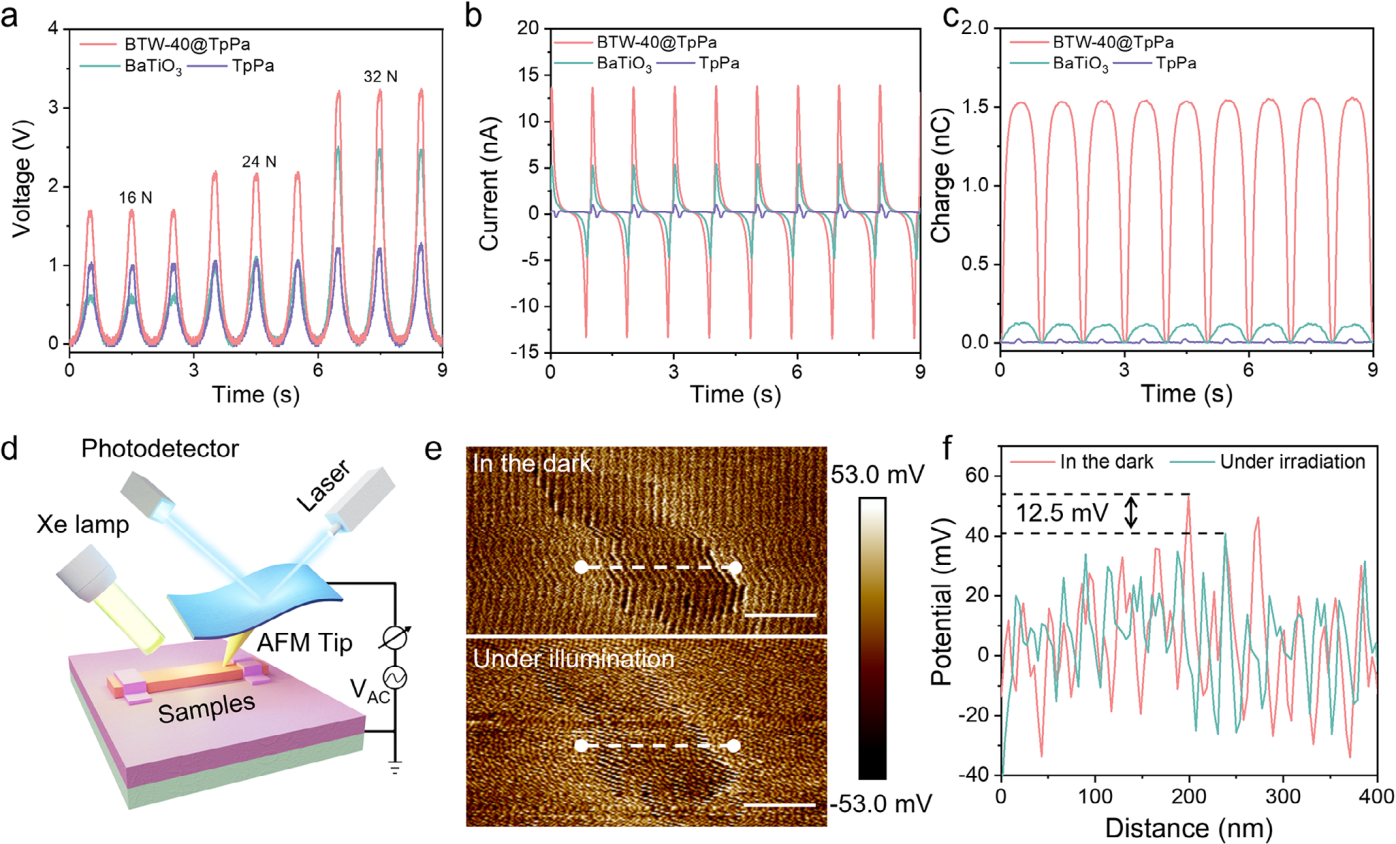
Piezoelectric response characterization. a) The piezoelectric output voltage of BTW‐40@TpPa under different applied forces (a constant frequency = 1 Hz). The piezoelectric output b) current and c) transfer charge of BTW‐40@TpPa under a pulsed force (Force = 32 N, frequency = 1 Hz). d) Schematic diagram of KPFM measurement device under light illumination (λ >420 nm). e) KPFM potential images of BTW‐40@TpPa in the dark and under illumination. The scale bars are 200 nm. f) The corresponding surface potential curves of BTW‐40@TpPa.

In general, the application of external stress induces lattice distortion in piezoelectric crystals, breaking cation‐anion charge symmetry to liberate bound surface charges and establish piezopotential gradients. To visually confirm the piezoelectric activity, a periodic force (Force = 16, 24, 32 N, frequency = 1 Hz) was applied to BaTiO_3_, TpPa, and BTW‐40@TpPa. As shown in **Figure** **5**a, the output open‐circuit voltage of BTW‐40@TpPa and BaTiO_3_ nanowires increased with the applied force, indicating significant piezoelectric signals. Notably, although TpPa exhibited a constant voltage output of ≈1 V, this value remained independent of the applied force. This behavior can be attributed to a triboelectric effect generated by the compaction of TpPa powders.^[^ ^34^ ^]^ Meanwhile, the piezoelectric output current and transferred charge were measured under a pulsed force (Force = 32 N, frequency = 1 Hz) and demonstrated remarkable improvements compared to those of BaTiO_3_ and TpPa. As illustrated in Figure 5b,c, the piezoelectric output current and transferred charge of BTW‐40@TpPa reached ≈13.74 nA and 1.53 nC, respectively. These values are 2.63 and 12.75 times higher than those of BaTiO_3_ and 13.52 and 59.86 times higher than those of TpPa. This enhancement arises from the robust interfacial interaction between BaTiO_3_ and TpPa within the heterojunction structure, which induces substantial polarization charges along the edges of the BaTiO_3_ nanowires. Additionally, the coupling mitigates the metallic‐state screening effect at the edges of BaTiO_3_.^[^ ^24^, ^35^ ^]^ These findings suggest that BTW‐40@TpPa can release abundant bound charges to generate a stronger piezopotential under the same compressive stress compared to BaTiO_3_ nanowires, thereby enabling directional migration and effective separation of electrons toward the catalytic surface.

Piezo‐response force microscopy (PFM) was employed to investigate the piezoelectric response of BTW‐40@TpPa before and after light irradiation. Upon application of ±5 V to the sample surface, a characteristic butterfly‐shaped amplitude hysteresis loop was observed, accompanied by an ≈180° phase angle shift (Figure S29, Supporting Information), confirming both a strong piezoelectric response and the occurrence of polarization switching. Moreover, vertical PFM amplitude scans under light irradiation exhibited brighter contrast compared to those measured in the dark (Figure S29a,d, Supporting Information), indicating an enhanced piezoelectric response upon illumination. The maximum effective piezoelectric coefficient *d*_33_ was quantitatively estimated from the slope of the PFM amplitude–voltage loops (Figure S29b,e, Supporting Information), yielding values of 280 pm V^−1^ in the dark and 305 pm V^−1^ under illumination. These results clearly demonstrate a positive effect of light irradiation on the piezoelectric performance of BTW‐40@TpPa. To further evaluate the real‐time piezo‐photo‐response of BTW‐40@TpPa, the Kelvin probe force microscopy (KPFM) was employed for in situ observation of surface potential under dark and light illumination (Figure 5d). The surface potential was measured using KPFM (Figure 5e). Under the mechanical stress applied by the probe tip, a positive surface potential was generated, reaching a maximum value of 53.2 mV in the dark, thereby confirming its piezoelectric properties. However, upon introducing light illumination, the maximum surface potential decreased to 40.7 mV (Figure 5f). This reduction can be attributed to the migration of photogenerated electrons and holes toward opposite sides driven by the piezopotential gradients. Subsequently, the electrons are partially neutralized by the positive piezoelectric charges, leading to a decrease in surface potential due to the charge shielding effect.^[^ ^28^, ^36^ ^]^ This process highlights the beneficial role of the piezopotential in promoting carrier separation and enhancing catalytic activity.

In order to further reveal the promotion of the piezoelectric effect on the reaction thermodynamics, the Gibbs free energy change (ΔG) of the intermediate states involved in the hydrogen evolution reaction (HER) is calculated by density functional theory (DFT). The HER likely proceeds via two single‐electron transfer steps driven by photogenerated electrons, leading to the formation of surface‐bound *H intermediates. According to previous studies,^[^ ^28^, ^37^ ^]^ the oxygen site in TpPa has been identified as a favorable active center for *H adsorption and coordination (Figure S30, Supporting Information). Figure S31 (Supporting Information) presents the elementary steps of the HER and the corresponding free energy profiles under different conditions—specifically, with and without light‐induced bias potential (U) or a polarized electric field, for all studied systems. At U = 0 V, the calculated ΔG values for HER on TpPa, BaTiO_3_, and BaTiO_3_@TpPa are 0.44, 0.28, and 0.17 eV, respectively. These values are notably higher than those obtained when considering the influence of the piezoelectric effect, which lowers the free energy barriers to 0.36, 0.23, and 0.12 eV, respectively. When a light‐induced external bias potential (U = 0.5 V) is applied to TpPa, BaTiO_3_, and BaTiO_3_@TpPa, the free energy changes become negative, indicating that the photocatalytic HER becomes thermodynamically spontaneous under visible light irradiation, particularly when combined with ultrasound. These DFT calculations confirm that the piezoelectric effect significantly enhances the HER activity of BaTiO_3_@TpPa by lowering the reaction barrier and facilitating charge transfer, thereby improving the overall H_2_ evolution performance.

### 2.4 Synergistic Mechanisms of Piezopotential and Energy Band Alignment

**Figure 6:**
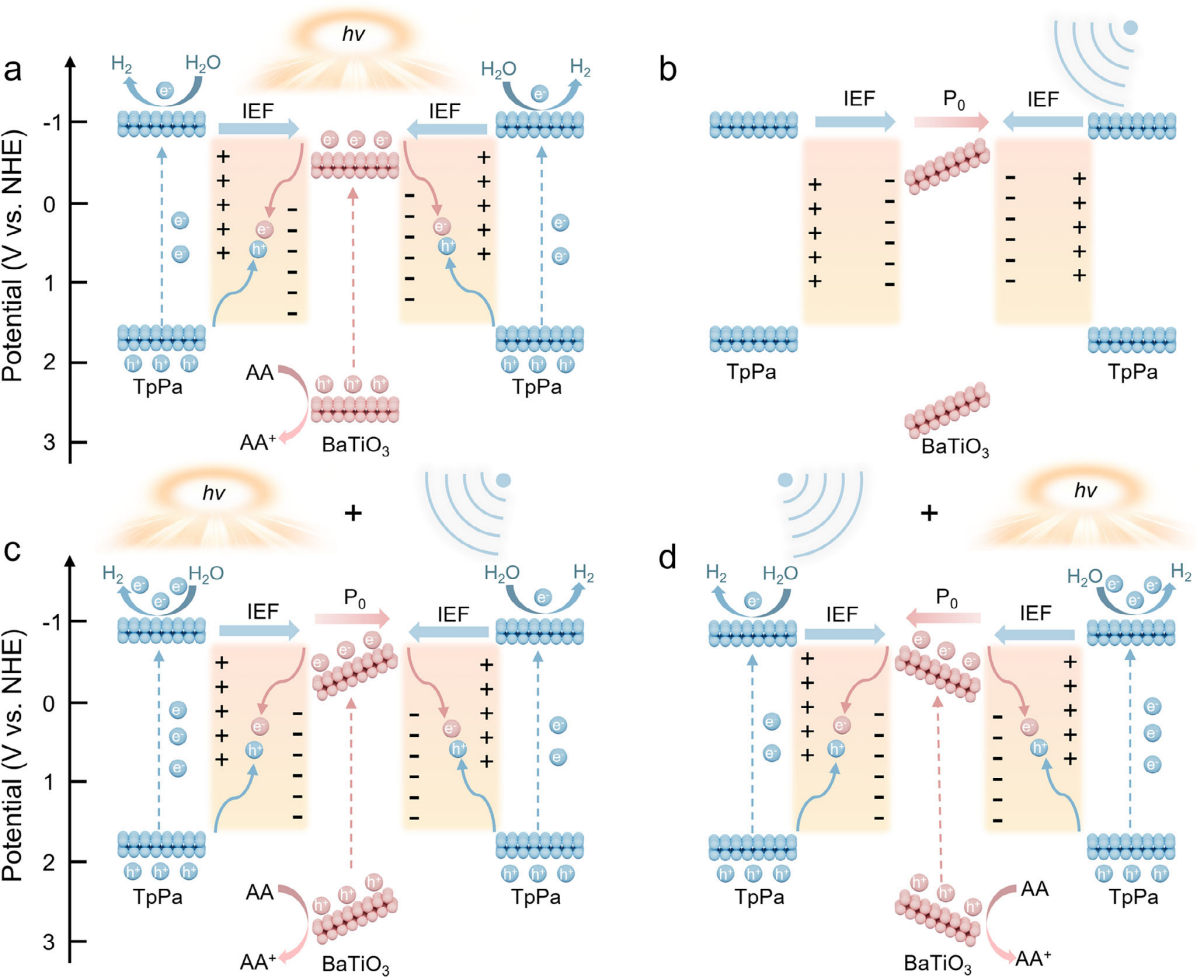
Schematic representation of electron transfer of a) photocatalysis, b) piezocatalysis, c,d) piezo‐photocatalysis with opposite direction of polarization field (P_0_).

In sight of the comprehensive experimental and characterization results, we propose the underlying enhancement mechanisms and the pivotal roles of the piezopotential and energy band alignment in accelerating electron transfer for the photoreduction of water molecules. Under the excitation of light illumination, the IEF drives the recombination of CB electrons from BaTiO_3_ with VB holes from TpPa, following *Z*‐scheme charge transfer pathways (**Figure** **6**a). Although parts can be consumed at the contact interface, the remaining VB holes in TpPa still recombine with the electrons within the bulk phase of TpPa due to insufficient transfer dynamics, thereby hindering the photoreduction reaction. When only ultrasound is applied to the reaction system, abundant bound polarization charges are released, inducing band bending^[^ ^38^ ^]^ and generating a polarization field (P_0_) within the bulk phase of the catalysts (Figure 6b). Nevertheless, the piezopotential may be limited by screening charge effects, which fall short of providing the Gibbs free energy required for the water redox reaction, resulting in suboptimal catalytic performance. In contrast, when both ultrasound and visible light irradiation are applied synergistically, the limitations of the redox piezopotential in piezocatalysis and carrier transfer dynamics in photocatalysis can be significantly overcome. As demonstrated in Figure 6c,d, the stress‐modulated polarization switching establishes unidirectional transport channels: under left‐to‐right polarization, electrons in the CB of BaTiO_3_ migrate counter‐field toward left interface while the holes in the VB of TpPa follow field direction to right interface, where they are consumed by electrons from BaTiO_3_. Conversely, when the P_0_ switches its direction (right‐to‐left), the holes of TpPa and the electrons of BaTiO_3_ still recombine at the contact interface. This ensures the effective separation of electrons and holes of TpPa within the bulk phase and maximizes electron utilization efficiency at the catalytic surface.

## 3 Conclusion

In conclusion, to address the limitations of insufficient piezopotential in piezocatalysis and inefficient carrier transfer dynamics in photocatalysis, we successfully synthesized BTW‐40@TpPa piezo‐photocatalysts with exceptional visible‐light harvesting and stress‐responsive capabilities. The energy band alignment and covalent bonding between TpPa and BaTiO_3_ facilitate the formation of *Z*‐scheme heterostructures, providing an efficient pathway for charge carrier transfer. Furthermore, the piezopotential generated by ultrasound serves as a strong driving force to enhance the separation and utilization efficiency of photoexcited electrons. This synergy results in a remarkable hydrogen evolution rate of 33 mmol g^−1^ h^−1^ and an outstanding apparent quantum yield of 9.39%, which are 4.56‐fold and 10.06‐fold higher than those achieved under pure photocatalytic conditions. Our study demonstrates the strategic integration of piezopotential and energy band engineering to design high‐efficiency catalysts, offering significant potential for energy conversion through synergistically leveraging solar and mechanical energy.

## 4 Experimental Section

### Synthesis of BaTiO_3_, APTES‐BaTiO_3_, and TpPa

The BaTiO_3_ nanowires were synthesized using a reported two‐step hydrothermal method, with titanium dioxide (TiO_2_) and barium hydroxide octahydrate (Ba(OH)_2_·8H_2_O) serving as precursors.^[^ ^22^, ^39^ ^]^ The amine‐functioned BaTiO_3_ nanowires were prepared in accordance with the reported procedures using 3‐aminopropyltriethoxysilane (APTES).^[^ ^11a^ ^]^ TpPa was prepared with 1,3,5‐triformylphloroglucinol (Tp) and p‐phenylenediamine (Pa) by the previously reported solvothermal process.^[^ ^25^ ^]^ Detailed information is in Supporting Information.

### Synthesis of TpPa‐Encapsulated BaTiO_3_ Nanowires

The synthesis of TpPa‐encapsulated BaTiO_3_ nanowires was similar to that of TpPa except that a certain mass of APTES‐BaTiO_3_ nanowires was added the solvothermal reaction systems. Specifically, Tp (31.5 mg, 0.15 mmol), Pa (24 mg, 0.225 mmol), and APTES‐BaTiO_3_ were weighed into a pyrex tube (o.d. × i.d. = 10 × 8 mm). Then, a co‐solvent system (1,4‐dioxane/mesitylene, 1:1 v/v, 1.5 mL total) was injected into the above mixtures, followed by sonication for 5 min to disperse thoroughly. An aqueous acetic acid catalyst (0.25 mL, 6 mol L^−1^) was introduced, with subsequent ultrasonication (5 min) to homogenize the ternary phase (aqueous/organic/solid). The tube was then frozen in a liquid nitrogen bath and flame‐sealed under dynamic vacuum, and thermally annealed at 120 °C for 72 h in oven. The obtained powder was washed by methanol and tetrahydrofuran to remove unreacted monomers, and then dried at 80 °C under vacuum overnight to give red TpPa‐encapsulated BaTiO_3_ nanowires. The different mass ratios of BaTiO_3_/(BaTiO_3_+TpPa), ranging from 20% to 60%, were synthesized and designated as BTW‐X@TpPa (X = 20, 40, 60). Here, the mass of TpPa denoted the summation of (Tp + Pa) without the consideration of yield.

### Piezo‐Photocatalytic H_2_ Production

The online system (Labsolar‐6A, Beijing Perfectlight Technology Co., Ltd., China) was used to investigate the piezo‐photocatalytic H_2_ evolution performance of the as‐synthesized catalyst. The light source was a 300 W Xe lamp with a 420 nm filter. A periodical stress was provided via an ultrasound cleaner (KQ‐300VDE, Kunshan Shumei Ultrasonic Instruments Co., Ltd., China) with an adjustable frequency of 45, 80, 100 kHz and a constant power of 300 W. A quartz flask was charged with 5 mg of catalysts, 0.1 mol L^−1^ ascorbic acid aqueous solution (160 ml) and 1 g L^−1^ H_2_PtCl_6_ (66 µL) as a cocatalyst. The resulting suspension was ultrasonicated until the photocatalyst was well‐dispersed. The dispersions were first illuminated for 30 min to complete 0.5 wt% Pt photo‐deposition. The reaction mixture was evacuated for 30 min to ensure complete removal of gases, and the pressure was below 1.1 kPa followed by the piezo‐photocatalytic reaction. The temperature of the reaction solution was maintained at 5 °C by circulation of cool water. The evolved gases were detected on an online gas chromatograph (GC9790, Zhejiang Fuli Instrument Co., Ltd., China) with a thermal conductive detector.

### Calculation of Apparent Quantum Yield (AQY)

The AQY was measured by a Xe lamp equipped with bandpass filters (λ  =  420, 500, 550 and 600 nm, respectively). The light intensity was measured using an optical power meter (PL‐MW2000, Beijing Perfectlight Technology Co., Ltd., China). For the AQY of piezo‐photocatalysis, the ultrasonic vibration (300 W, 45 kHz) was introduced into the photocatalytic system. The AQY was calculated using the equation:

(1) $$\begin{array}{cc} AQY & =\frac{N_{e}}{N_{P}}\times 100\%=\frac{2\times M\times N_{A}}{\frac{E_{total}}{E_{photon}}}\times 100\%=\frac{2\times M\times N_{A}}{\frac{\left(S\times P\times t\right)}{\left(h\times \frac{c}{\lambda }\right)}}\times 100\%\\ & =\frac{2\times M\times N_{A}\times h\times c}{S\times P\times t\times \lambda }\times 100\% \end{array}$$

where *M* is the amount of H_2_ (mol), *N_A_* is the Avogadro constant (6.022×10^23^ mol^−1^), *h* is the Planck constant (6.626×10^−34^ J s), *c* is the speed of light (3×10^8^ m s^−1^), *S* is the irradiation area (m^2^), *P* is the intensity of irradiation light (W cm^−2^), *t* is the photoreaction time (s), *λ* is the wavelength of the monochromatic light (m).

## Conflict of Interest

The authors declare no conflict of interest.

## Supporting information



Supporting Information

Supplemental Video 1

## Data Availability

The data that support the findings of this study are available from the corresponding author upon reasonable request.
