# Supplementary material for: Piezoceramics Boosting Photocatalytic Hydrogen Evolution of Covalent Organic Frameworks
Source: Adv Sci (Weinh). 2025 Jul 11;12(38):e08062. doi: 10.1002/advs.202508062 (PMC12520523; doi:10.1002/advs.202508062)
Supplement: Supplementary file 1 — Supporting Information [file ADVS-12-e08062-s002.docx]

**Supporting Information**

**Piezoceramics Boosting Photocatalytic Hydrogen Evolution of Covalent Organic Frameworks**

*Jinglin Gao, Jianyu Xiao, Shijia Luo, Xuzheng Ji, Congcong Yin, Yuping Wu, Xin Zhao, and Yong Wang**

School of Energy and Environment, Southeast University, Nanjing, 211189, Jiangsu, P. R. China.

*E-mail: [yongwang@seu.edu.cn](file:///D:\\DESKTOP\\01汪勇\\COF-EDA%20synthesis\\yongwang@seu.edu.cn) (Y. W.)

1. **Materials**

1,3,5-Triformylphloroglucinol (Tp, 98%), p-phenylenediamine (Pa, 99%), benzidine (BD, 98%) were sourced from Jilin Chinese Academy of Sciences–Yanshen Technology and Aladdin, respectively. Titanium dioxide (TiO_2_, 99%), barium hydroxide octahydrate (Ba(OH)_2_·8H_2_O, 98%) and 3-aminopropyltriethoxysilane (APTES, 98%) were purchased from Sigma-Aldrich. Sodium hydroxide (NaOH, 97%), hydrochloric acid (HCl, 36~38%), glacial acetic acid (99.5%), mesitylene (97%), 1,4-dioxane (99%), ethanol (AR), tetrahydrofuran (99.5%) and methanol (99.5%) were supplied by Sinopharm Chemical Reagent. Water was obtained by an ultrapure water system (PLUS-F2-20TF, Nanjing EPED Technology Co., Ltd.). High-purity Ar (99.999%) was provided from local suppliers. All chemicals were used without further purification.

1. **Sample preparation**
   1. **Synthesis of BaTiO_3_ nanowires**

The synthesis of BaTiO_3_ nanowires was accomplished through a reported two-step hydrothermal reaction with slight modifications^[1]^. First, high aspect ratio sodium trititanate (Na_2_Ti_3_O_7_) nanowires were synthesized. Typically, 1.45 g of TiO_2_ powder was mixed with 30 ml of NaOH aqueous solution (10 mol L^-1^). The mixture was stirred at 400 rpm for 30 minutes. Then, the mixed solution was transferred into a 50 ml Teflon-lined autoclave. The suspension was sealed in a stainless-steel autoclave at 200°C for 72 hours. After the autoclave was cooled to room temperature, the obtained Na_2_Ti_3_O_7_ nanowires were washed with water then soaked in diluted 0.2 mol L^-1^ HCl aqueous solution for 4 hours to yield hydrogen trititanate (H_2_Ti_3_O_7_) nanowires. Then, the resulting powder was washed with water four times through centrifugation and subsequently dried on a vacuum oven at 60°C overnight. The BaTiO_3_ nanowires were synthesized from H_2_Ti_3_O_7_ nanowires the by a second hydrothermal reaction in Ba(OH)_2_·8H_2_O aqueous solution. Specially, 0.075 g H_2_Ti_3_O_7_ nanowires were was mixed with 30 ml of Ba(OH)_2_·8H_2_O aqueous solution (0.02 mol L^-1^). Then, the mixed solution was transferred into a 50 ml Teflon-lined autoclave. The suspension was sealed in a stainless-steel autoclave at 210°C for 5 hours. After the autoclave was cooled to room temperature, the obtained BaTiO_3_ nanowires were washed with water and ethanol three times and dried at 60°C vacuum oven overnight.

**2.2 Synthesis of TpPa via solvothermal method**

TpPa was synthesized by the previously reported solvothermal process^[2]^. Specifically, a pyrex tube (o.d. × i.d. = 10 × 8 mm) was charged with Tp (63 mg, 0.3 mmol), Pa (48 mg, 0.45 mmol), 1,4-dioxane (1.5 mL) and mesitylene (1.5 mL), which were mixed by sonicating for 5 min. Subsequently, the acetic acid aqueous solution (0.5 mL, 6 mol L^-1^) as the catalyst was added in the above-mentioned solution. After sonicating for another 5 min, the tube was flash frozen at 77 K in a liquid nitrogen bath and sealed under vacuum. The reaction mixture was heated at 120°C for 3 days. The obtained powder was washed by methanol and tetrahydrofuran, and then dried at 80°C under vacuum overnight to give red TpPa.

**2.3 Synthesis of APTES-BaTiO_3_ nanowires**

BaTiO_3_ nanowires (1 g) were ultrasonically dispersed into ethanol (100 mL) for 20 minutes. A solution of APTES (1 mL) and ethanol (20 mL) was slowly added to the above solution under vigorous stirring (1000 rpm) at 60°C for 5 hours. Subsequently, the samples were centrifuged and washed three times with distilled water and ethanol. Moreover, the obtained APTES-BaTiO_3_ samples were dried at 60°C overnight.

**2.4 Synthesis of TpBD-encapsulated BaTiO_3_ Nanowires**

The synthesis of TpBD-encapsulated BaTiO_3_ nanowires was similar to that of BaTiO_3_@TpPa except that 0.45 mmol Pa was replaced by 0.225 mmol BD in the solvothermal reaction systems. Specifically, Tp (31.5 mg, 0.15 mmol), BD (41.4 mg, 0.225 mmol) and 48.6 mg APTES-BaTiO_3_ was weighed into a pyrex tube (o.d. × i.d. = 10 × 8 mm). Then, a co-solvent system (1,4-dioxane/mesitylene, 1:1 v/v, 1.5 mL total) was injected into the above mixtures, followed by sonication for 5 minutes to disperse thoroughly. An aqueous acetic acid catalyst (0.25 mL, 6 mol L^-1^) was introduced, with subsequent ultrasonication (5 min) to homogenize the ternary phase (aqueous/organic/solid). The tube was then frozen in a liquid nitrogen bath and flame-sealed under dynamic vacuum, and thermally annealed at 120°C for 72 h in oven. The obtained powder was washed by methanol and tetrahydrofuran to remove unreacted monomers, and then dried at 80°C under vacuum overnight to give orange TpBD-encapsulated BaTiO_3_ nanowires.

**2.5 Synthesis of TpPa-encapsulated TiO_2_ nanoparticles**

The synthesis of TpPa-encapsulated TiO_2_ nanoparticles was similar to that of BaTiO_3_@TpPa except that APTES-BaTiO_3_ was replaced by APTES-TiO_2_ in the solvothermal reaction systems. Specifically, Tp (31.5 mg, 0.15 mmol), Pa (24 mg, 0.225 mmol) and 37 mg APTES-TiO_2_ was weighed into a pyrex tube (o.d. × i.d. = 10 × 8 mm). Then, a co-solvent system (1,4-dioxane/mesitylene, 1:1 v/v, 1.5 mL total) was injected into the above mixtures, followed by sonication for 5 minutes to disperse thoroughly. An aqueous acetic acid catalyst (0.25 mL, 6 mol L^-1^) was introduced, with subsequent ultrasonication (5 min) to homogenize the ternary phase (aqueous/organic/solid). The tube was then frozen in a liquid nitrogen bath and flame-sealed under dynamic vacuum, and thermally annealed at 120°C for 72 h in oven. The obtained powder was washed by methanol and tetrahydrofuran to remove unreacted monomers, and then dried at 80°C under vacuum overnight to give red TpPa-encapsulated TiO_2_ nanoparticles.

1. **Characterization**

The morphologies and compositions of samples were observed by scanning electron microscope (SEM, S4800, Hitachi, Japan) at an accelerating voltage of 5 kV and the transmission electron microscopy (TEM, JEM-F200, JEOL, Japan) at a voltage of 200 kV. Powder X-ray diffraction (PXRD, Smart Lab SE, Rigaku, Japan) were performed to obtain crystal structure of materials with Cu-Kα radiation (λ = 0.15406 nm) at a scanning rate of 5° min^-1^. Fourier transform infrared (FT-IR, Nicolet 8700, Thermo Scientific, USA) spectra of the samples were obtained at the range of 4000-400 cm^-1^. Thermogravimetric analyses (TGA 2, METTLER TOLEDO, Switzerland) were conducted to analyze mass ratio of composites by heating samples at 10°C min^−1^ from 30 to 800°C in a nitrogen atmosphere. UV-vis spectrophotometer equipped with an integrating sphere (UV-2600i, Shimadzu, Japan) was performed to obtain the absorbance and reflectance spectra. X-ray photoelectron spectroscopy (XPS, Escalab 250Xi, Thermo Scientific, USA) measurements were carried out to analyze chemical compositions and valence state of elements. The ultraviolet photoelectron spectroscopy (UPS, Escalab 250Xi, Thermo Scientific, USA) of samples were also measured in order to confirm the [energy band structure](https://www.sciencedirect.com/topics/materials-science/electronic-band-structure). From UPS spectra, the [work function](https://www.sciencedirect.com/topics/physics-and-astronomy/work-function) (W_F_) of sample was calculated according to the equation W_F_ = hν–(E_Fermi_–E_cutoff_), in which hν is the He I energy (21.22 eV). Apparent surface areas were measured by nitrogen adsorption at 77 K using a volumetric adsorption analyzer (BELSORP MAX-X, Microtrac, Japan). The samples were degassed offline under vacuum at 120ºC for 24 h. The specific surface areas were evaluated using the BET model. Piezoelectric properties and ferroelectric properties of samples were characterized with a commercial atomic force microscope (AFM, Cypher S, Oxford Instruments, UK) equipped with a piezoresponse force microscopy (PFM) mode. The Kelvin probe force microscopy (KPFM) measurements were carried out by an atomic force microscope (AFM) with an AFM tip (Dimension Icon, Bruker, USA). Steady photoluminescence (PL) emission spectra and transient time resolved PL decay were obtained by luminescence spectrophotometer (FluoroMax-4, HORIBA Scientific, Japan) with a 398 nm excitation wavelength.

1. **Electrochemical measurements**

The electrochemical impedance, Mott-Schottky plots and transient current were measured via a CHI 660e (Chenhua Co. Lt., Shanghai, China) electrochemical station in a standard three-electrode configuration, using 0.5 M Na_2_SO_4_ as the electrolyte, sample-coated FTO glass as the working electrode, Pt wire as the counter electrode and Ag/AgCl electrode as the reference electrode. For the preparation of the working electrodes, 5 mg samples were added into 100 μL 5% Nafion and 0.5 mL ethanol/H_2_O (1:1) mixed solution, and ultrasonicated for 5 min. The catalyst suspension (100 μL) was dropped onto FTO glass (1 cm×1 cm), forming a film after drying naturally for 24 h. The impedance was recorded from 0.01 Hz to 100 kHz under visible light irradiation (300 W Xe lamp, λ > 420 nm) or ultrasonic vibration (300 W, 45 kHz). The transient current*-*time curve responses were recorded at five periodic on-off cycles under a constant bias of 0.5 V vs. Ag/AgCl reference electrode, following the exposure of the sample to visible light irradiation or ultrasonic vibration. The impedance-potential model was employed to collect the Mott-Schottky plots with the frequency of 1000, 2000 and 5000 Hz.

1. **Transient absorption spectroscopy measurements**

Femtosecond transient absorption spectroscopy (TAS) was performed using a Helios (Ultrafast) pump-probe setup integrated with a Coherent regenerative amplified laser system. The light source was a Ti:sapphire amplifier (Astrella, Coherent), delivering 800 nm pulses with a 100 fs duration, 1 kHz repetition rate, and pulse energy of 7 mJ. The output beam was divided using a beam splitter: one portion was directed to an optical parametric amplifier (TOPAS, Coherent) for generating pump pulses at 800 nm, while the other was focused onto sapphire and YAG crystals to produce a white-light supercontinuum probe, covering the 750–1600 nm spectral ranges. A motorized optical delay line enabled temporal scanning up to 8 ns between the pump and probe pulses. The pump beam was modulated at 500 Hz using a mechanical chopper and co-focused with the probe on the sample. The transmitted probe light was collimated and fed into a fiber-coupled multichannel spectrometer equipped with a CCD detector.

1. **Piezoelectric output measurements**

The BaTiO_3,_ TpPa, and BaTiO_3_@TpPa was placed into a mold and pressed it into a dense disc with a diameter of 13 mm to ensure that the sample had certain mechanical strength and uniformity. Then, polyimide tapes were used to encapsulate the sample to prevent it from breaking during testing. The top and bottom surfaces of the samples were attached using the copper electrodes and connected by wires. The outside of copper electrodes was also encapsulated using polyimide tapes to leakage of electricity. The tapes also attached the contact surface of stress equipment to friction and electrical interference. A linear motor (R-LP3, Nano Energy Instrument, China) was used to achieve contact separation movement under different force (16, 24, 32 N) and a constant frequency (1 hZ). The output voltage, current and transferred charge signals of the samples were tested using an oscilloscope (DSO-X 3024T, KEYSIGHT, USA) and an electrometer (Keithley 6514, Tektronix, USA). All measurements were conducted within a Faraday cage, thereby minimizing the influence of extraneous environmental noise.

1. **Detailed simulation methods**

**7.1 Finite element analysis**

Simulations based on the three-dimensional commercial Finite Element Method (FEM)^[3]^. The BaTiO_3_ with different morphologies and size was established by a three-dimension and a two-dimensional axisymmetric model in a region of surrounding water (relative permittivity = 80). The piezoelectric potential distributions of the BaTiO_3_ are calculated via the coupling between the Electrostatics Interface and the Solid Mechanics Interface in COMSOL Multiphysics 5.6. The bottom of BaTiO_3_ is fixed and grounded. Ultrasonic cavitation effect can initiate generate extremely high transient pressures (100 MPa) in vicinity of BaTiO_3_^[4]^. A 10^8^ N m^-2^ of boundary loading is applied to the surrounding of BaTiO_3_ to simulate ultrasonic stimulation. The piezoelectric effect can be expressed by the following equation:

$$\begin{matrix} \text{T=c}_{\text{E}}\text{S}-\text{e}^{\text{T}}\text{E} \\ \text{D=eS}+\varepsilon_{0}\varepsilon_{\text{rS}}\text{E} \end{matrix}$$

Where *T* and *S* are stress and strain vectors; *D* and *E* represent the electric displacement and the electric field vector respectively; *ε*_0_ denotes the vacuum permittivity constant. The other material parameters used in the simulation, such as density (*ρ* = 6020 kg m^-3^), elasticity matrix (*c*_E_), coupling matrix (e), and relative permittivity (*ε*_rS_) of BaTiO_3_ are available in the built-in database of COMSOL software. The main parameters are listed as follows:

$$\text{c}_{\text{E}}=\left[\begin{matrix} 2.75121 & 1.78967 & 1.51555 & 0 & 0 & 0 \\ 1.78967 & 2.75121 & 1.51555 & 0 & 0 & 0 \\ 1.51555 & 1.51555 & 1.6486 & 0 & 0 & 0 \\ 0 & 0 & 0 & 0.543478 & 0 & 0 \\ 0 & 0 & 0 & 0 & 0.543478 & 0 \\ 0 & 0 & 0 & 0 & 0 & 1.13122 \end{matrix}\right]\times10^{11}Pa$$

$$e=\left[\begin{matrix} 0 & 0 & 0 & 0 & 21.3043 & 0 \\ 0 & 0 & 0 & 0 & 0 & 0 \\ -2.69289 & -2.69289 & 3.65468 & 21.3043 & 0 & 0 \end{matrix}\right]C\cdot m^{-2}$$

$$\varepsilon_{rS}=\left[\begin{matrix} 1976.8 & 0 & 0 \\ 0 & 1976.8 & 0 \\ 0 & 0 & 111.7 \end{matrix}\right]$$

**7.2 Density functional theory**

All density functional theory (DFT) calculations were systematically executed using the Vienna Ab Initio Simulation Package (VASP) with the Perdew-Burke-Ernzerhof (PBE) generalized gradient approximation (GGA). We employed the projector-augmented wave (PAW) method to model core-electron interactions, expanding valence electrons in a plane-wave basis set with a 500 eV kinetic energy cutoff. Computational parameters were rigorously controlled: Gaussian smearing (0.05 eV width) governed partial orbital occupancies, while electronic and geometric convergence thresholds were set at 10^-4^ eV and 0.04 eV/Å, respectively. Dispersion forces were explicitly accounted for through Grimme's DFT-D3 methodology. For hydrogen diffusion analysis, the climbing image nudged elastic band (CI-NEB) method was implemented to determine H₂ migration energy barriers with atomic precision. Adsorption energetics were quantified using the fundamental relationship E_ads_ = E_ad/sub_ - E_ad_ - E_sub_, where E_ad/sub_, E_ad_ and E_sub_ are the optimized adsorbate/substrate system, the adsorbate in the structure and the clean substrate respectively. To simulate realistic surface conditions, structural relaxations preserved bulk-like characteristics by constraining the bottom three atomic layers throughout the optimization process.

The adsorption of hydrogen on surface was calculated as follows:

∆G(H*) = E (slab + H*) – E (slab) – 1/2E (H_2_) + ΔE (ZPE) – TΔS

Where E (slab + H*) is the total energy for the adsorption H, and E (slab) is the energy of pure surface, and E (H_2_) is the energy of H_2_ in gas phase. ΔE (ZPE) is the zero-point energy change and ΔS is the entropy change. the Gibbs free energy calculation was corrected by a constant of 0.24 eV at 298 K as:

∆G(H*) = E (slab + H*) – E (slab) – 1/2E (H_2_) + 0.24”

1. **Supporting Figures**


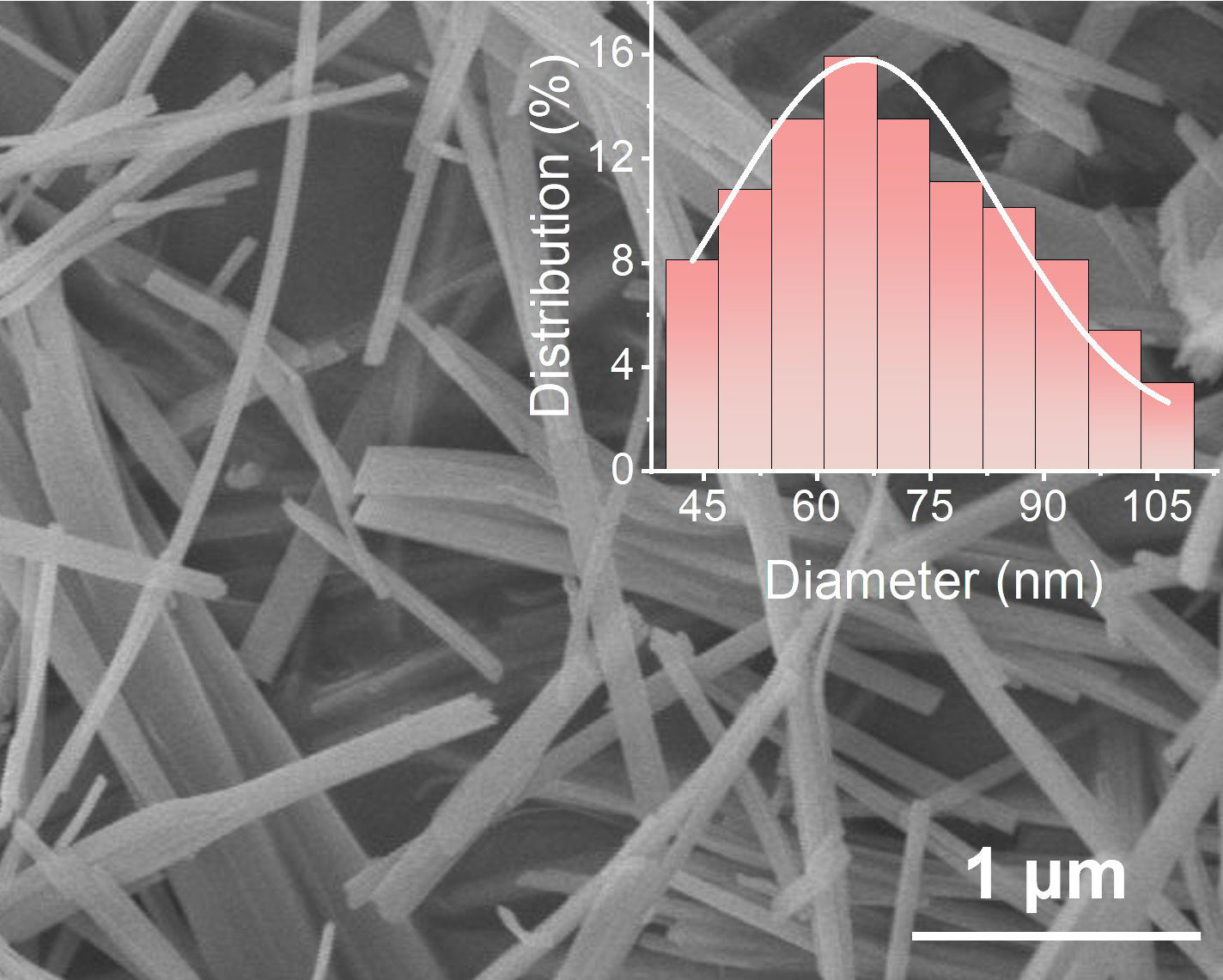


**Figure S1.** The morphology of BaTiO_3_ nanowires. Insets are the distribution of diameter of nanowires.


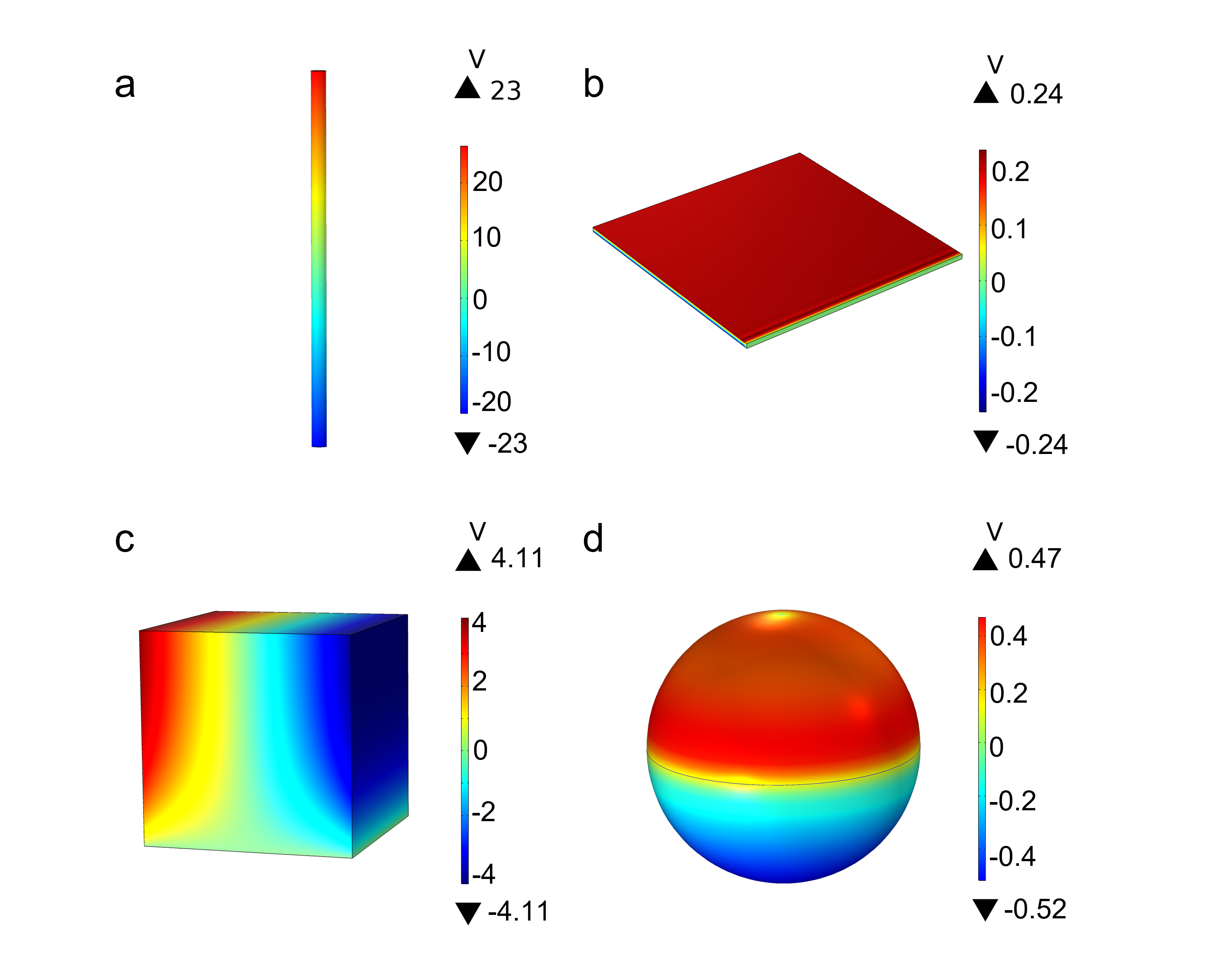


**Figure S2.** Finite element analysis of surface potential of BaTiO_3_ with different morphology by COMSOL Multiphysics 5.6. (**a**) Nanowires with lengths of 5 μm and diameters of 100 nm. (**b**) Nanosheets with lengths of 5 μm, widths of 5 μm and heights of 100 nm. (**c**) Nanocubes with lengths, widths and heights of 5 μm. (**d**) Nanospheres with diameters of 5 μm.


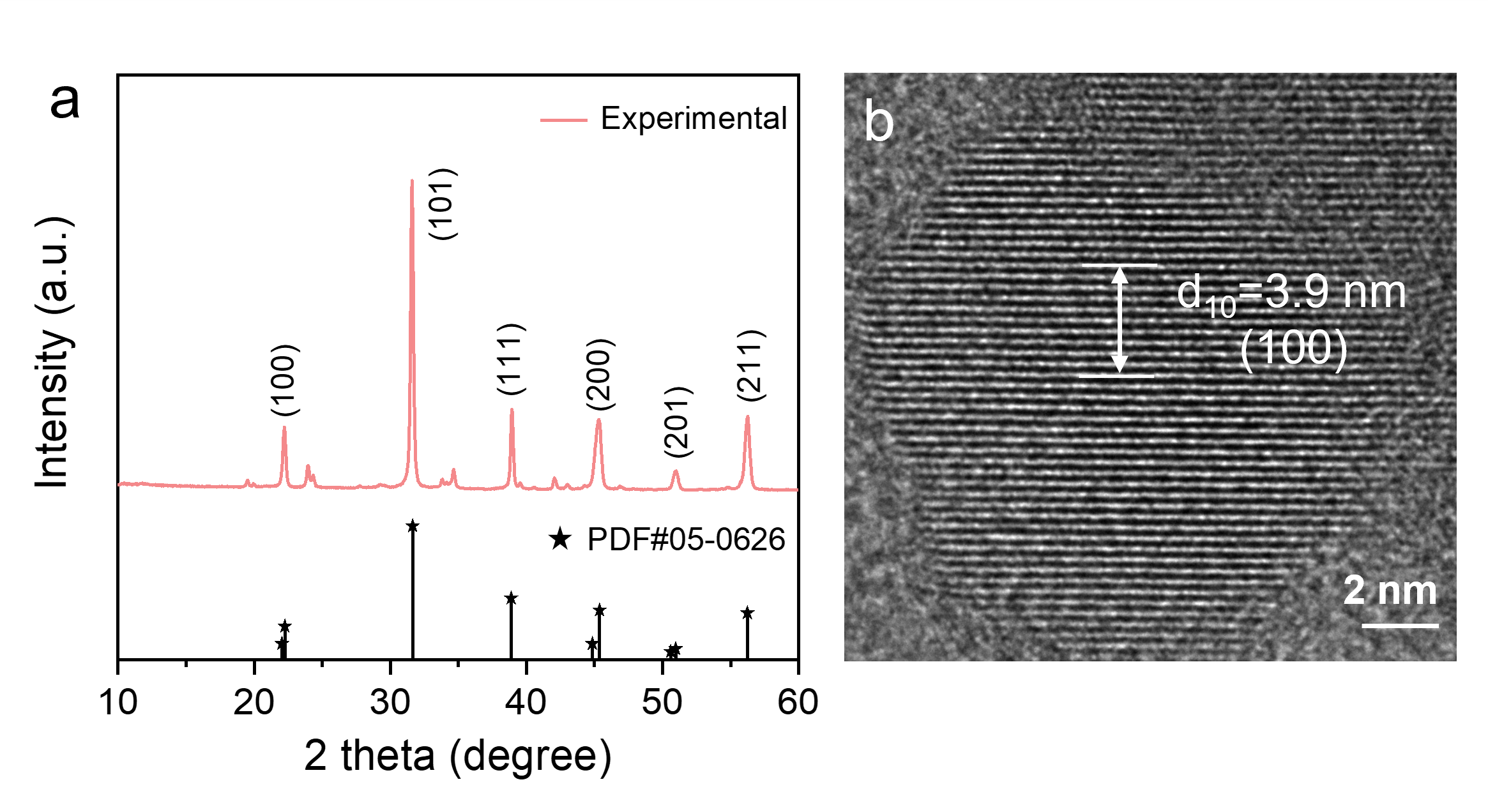


**Figure S3.** (**a**) Experimental PXRD and corresponding JCPDS patterns of BaTiO_3_, and (**b**) HRTEM image of BaTiO_3_ nanowires.


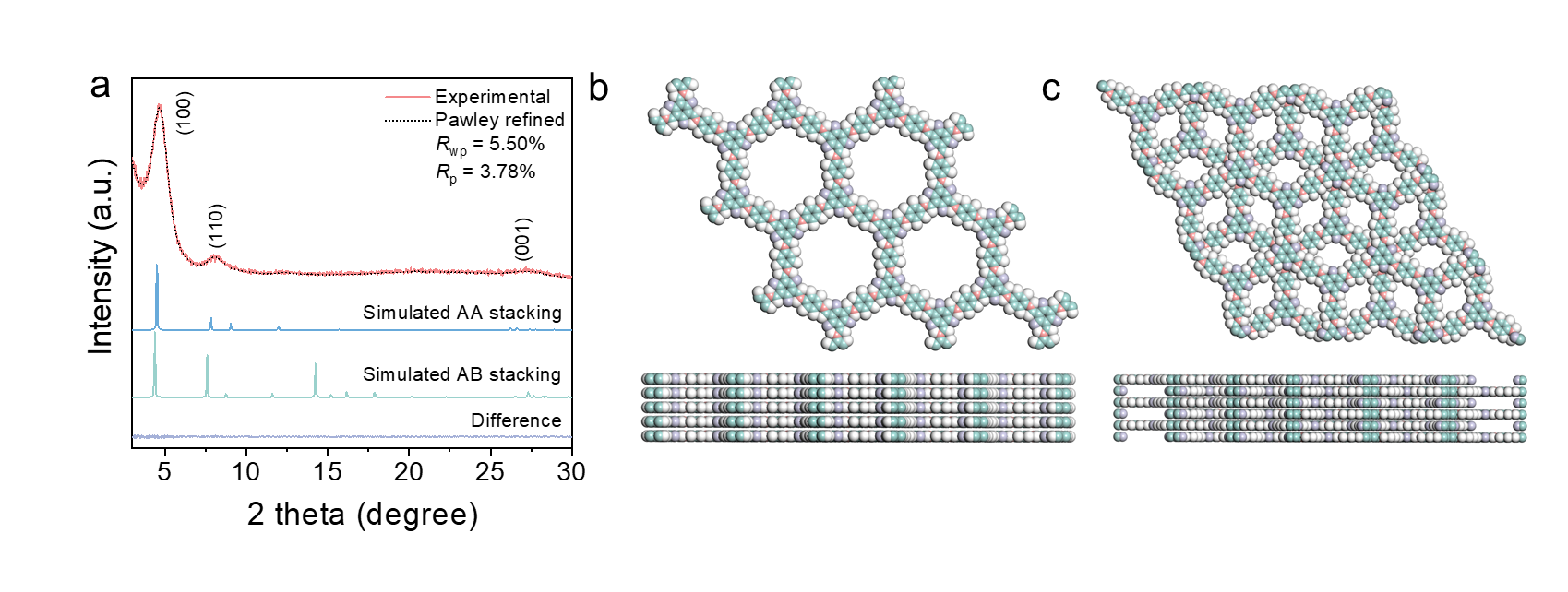


**Figure S4.** (**a**) Experimental and simulated PXRD patterns of TpPa. The top and side views of (**b**) eclipsed AA-stacking and (**c**) staggered AB-stacking models for TpPa.


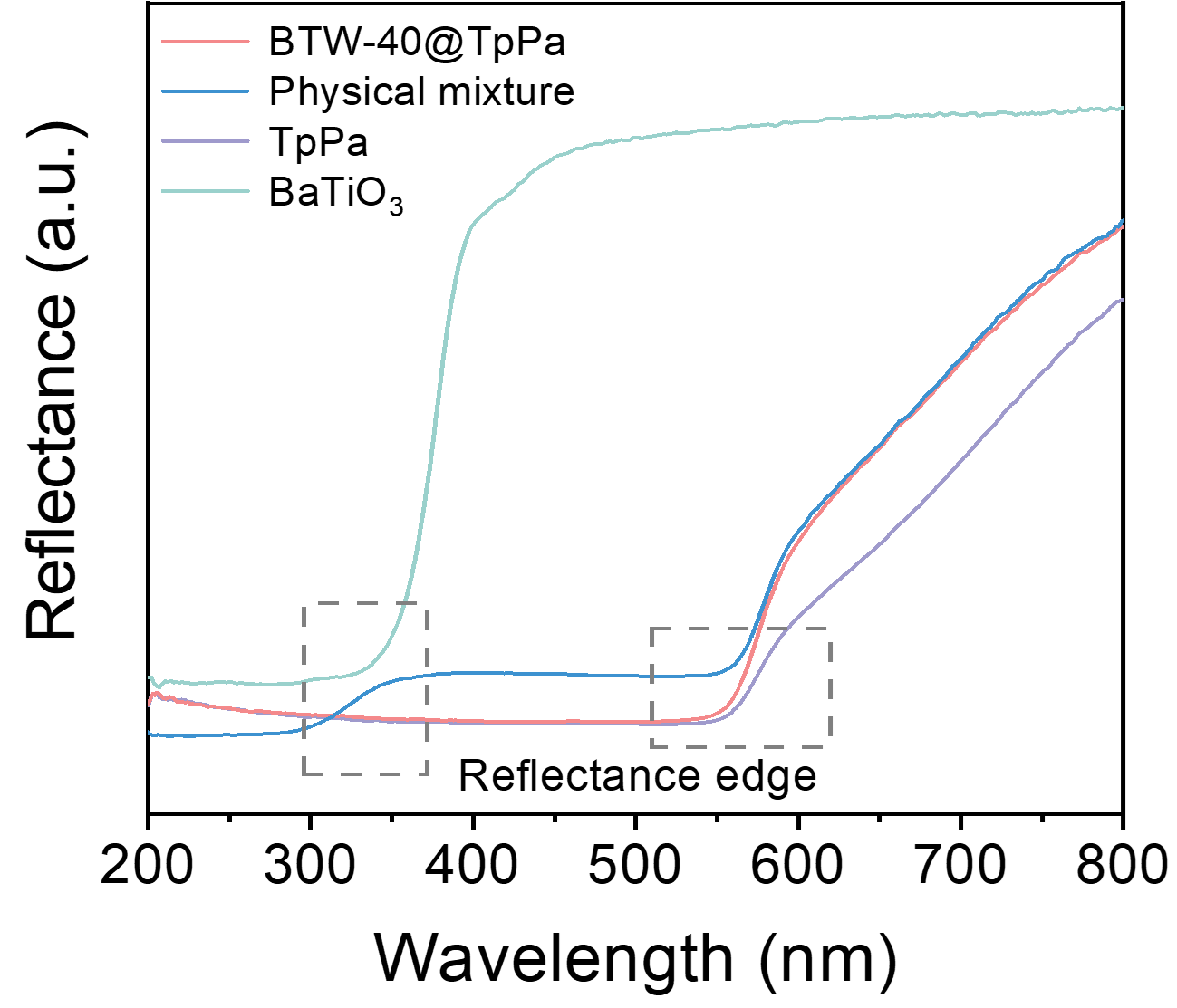


**Figure S5.** UV–vis DRS spectra of BaTiO_3_, TpPa, BTW-40@TpPa, and physical mixture.


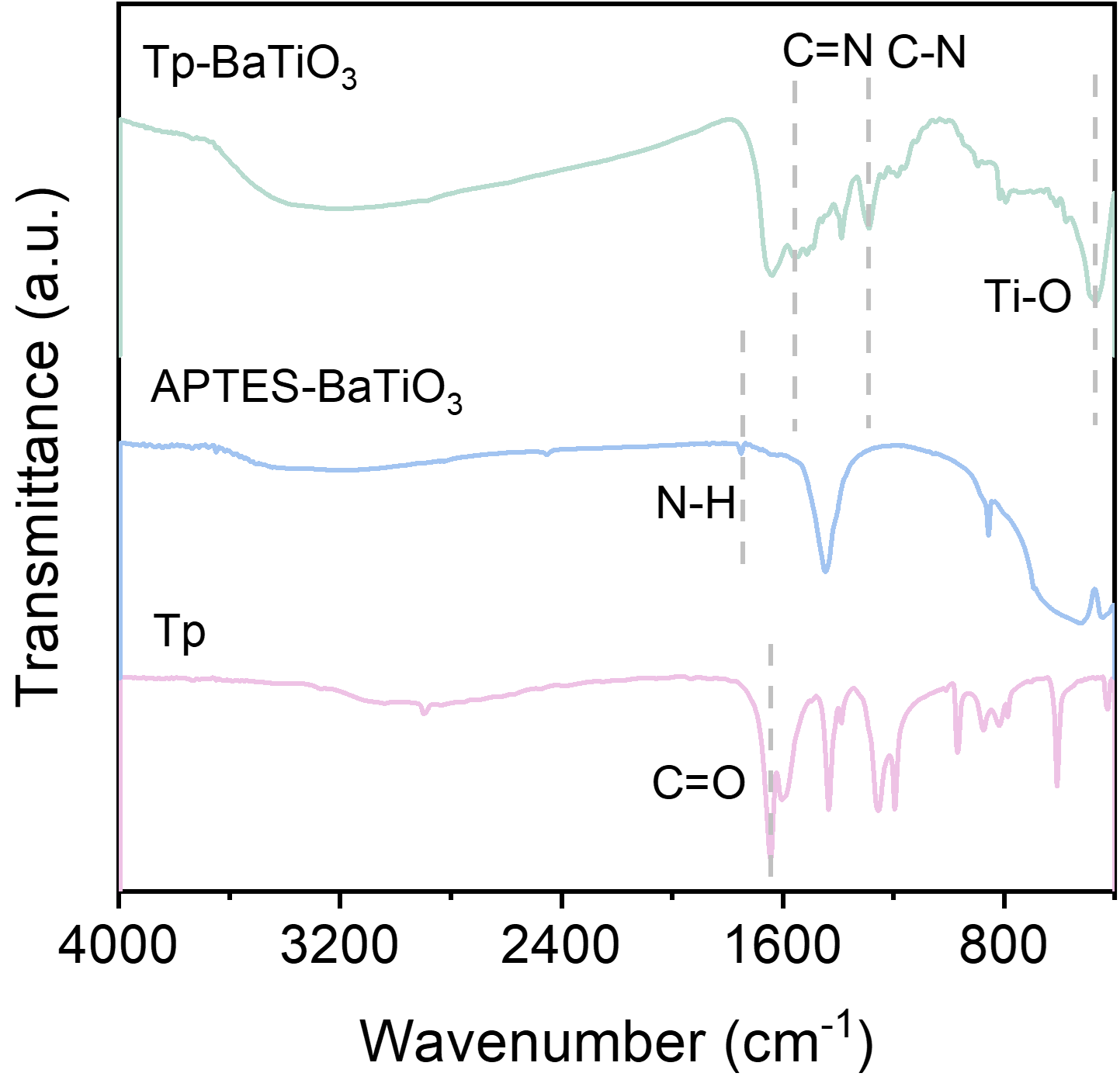


**Figure S6.** FT-IR spectra of APTES-BaTiO_3_, Tp, and Tp-BaTiO_3_. Compared with the APTES-BaTiO_3_, the spectrum of Tp-BaTiO_3_ shows two additional peaks at 1561 and 1281 cm^−1^, which are attributed to C=N and C-N, respectively. It is noteworthy that the peak at 1742 cm^-1^ from N-H groups of ATPES-BaTiO_3_ disappeared in the FT-IR spectrum of Tp-BaTiO_3_, which further demonstrates Tp has been successfully grafted on the surface of BaTiO_3_ by covalent bonds.


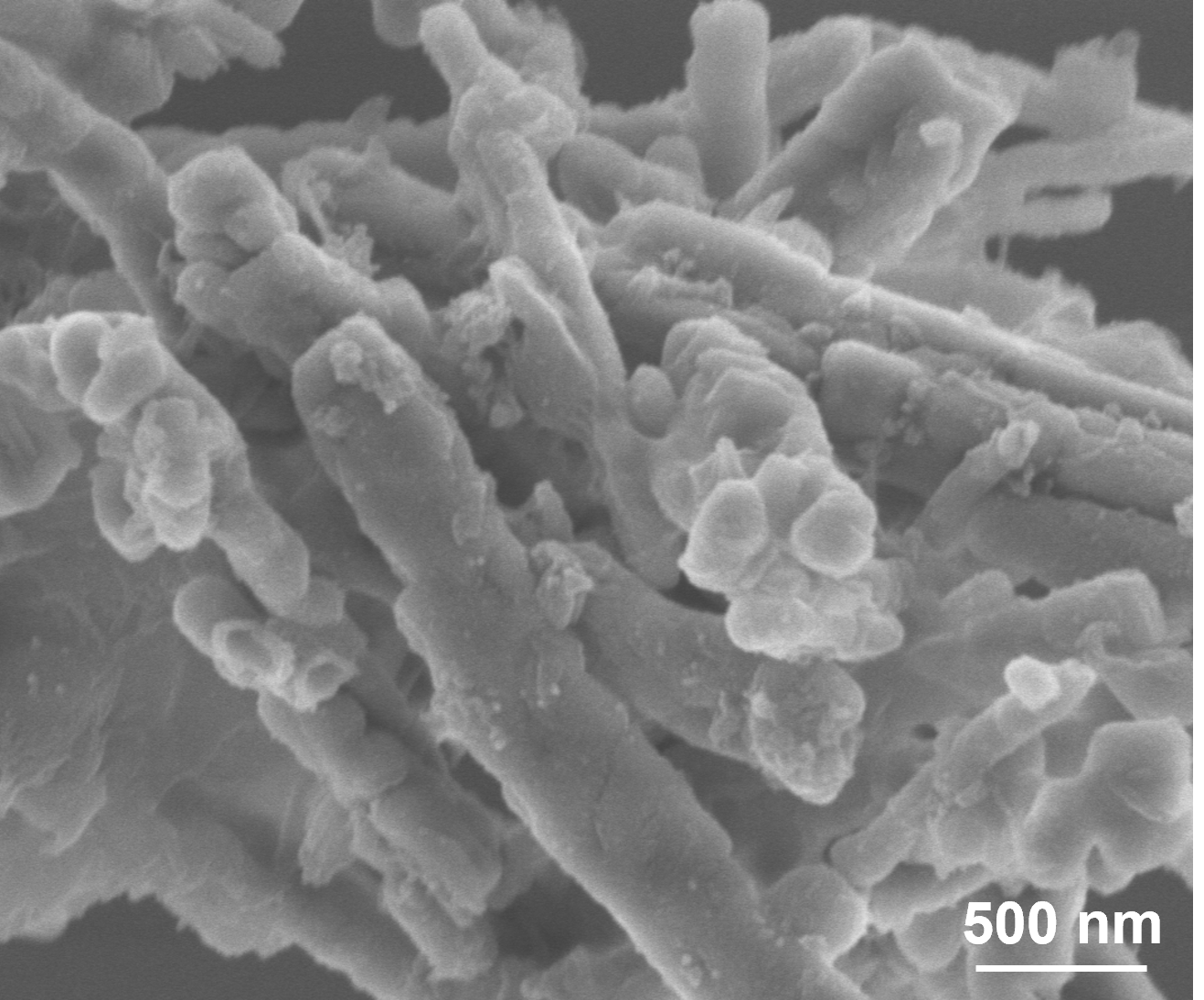


**Figure S7.** The SEM image of BTW-40@TpPa. The surface of BTW-40@TpPa shows a rougher morphology than that of pristine BaTiO_3_ nanowires, suggesting a successful encapsulation of TpPa.


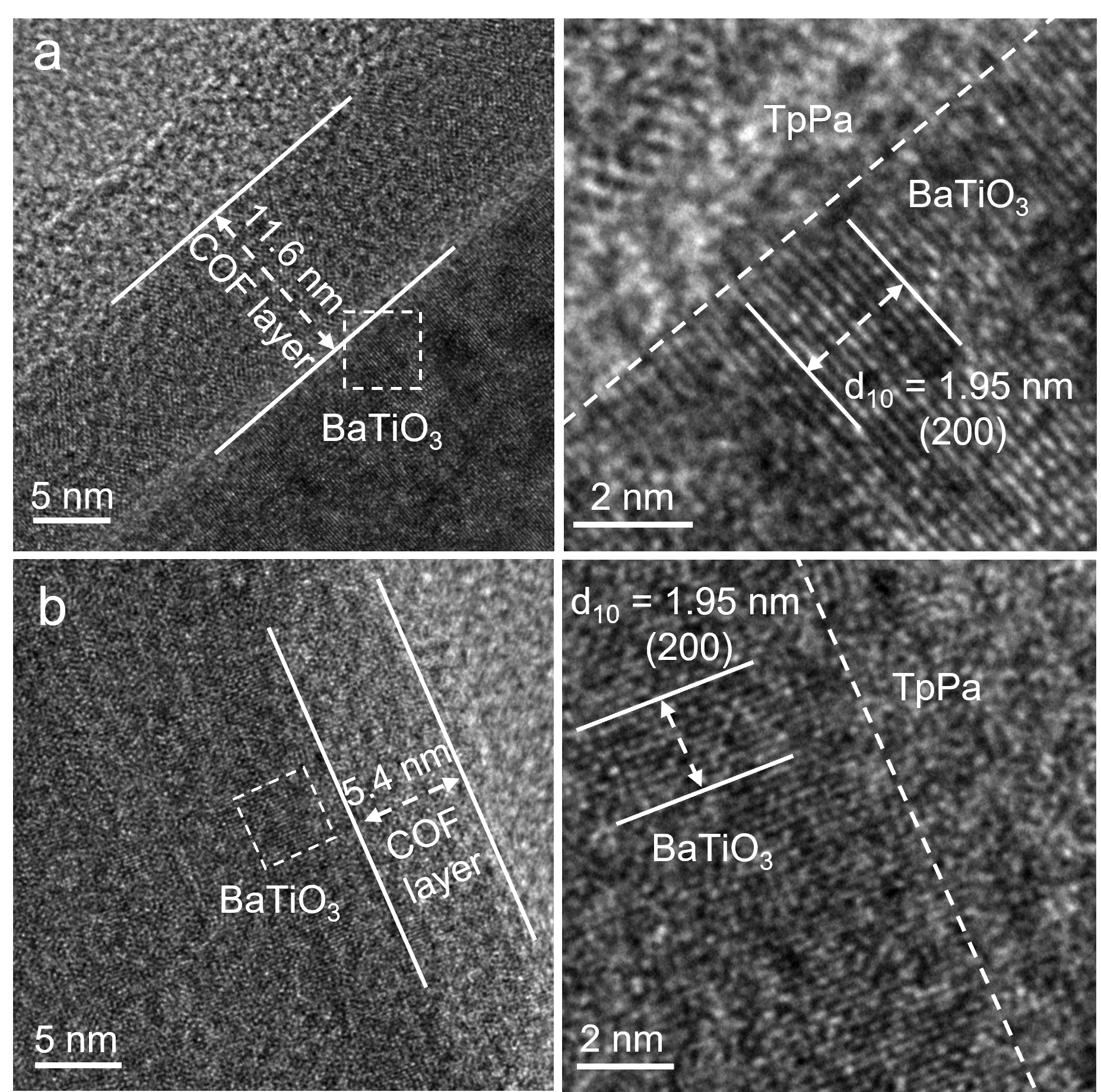


**Figure S8.** The HRTEM images of (**a**) BTW-20@TpPa and (**b**) BTW-60@TpPa. The images clearly show the thickness of shell layers of TpPa.


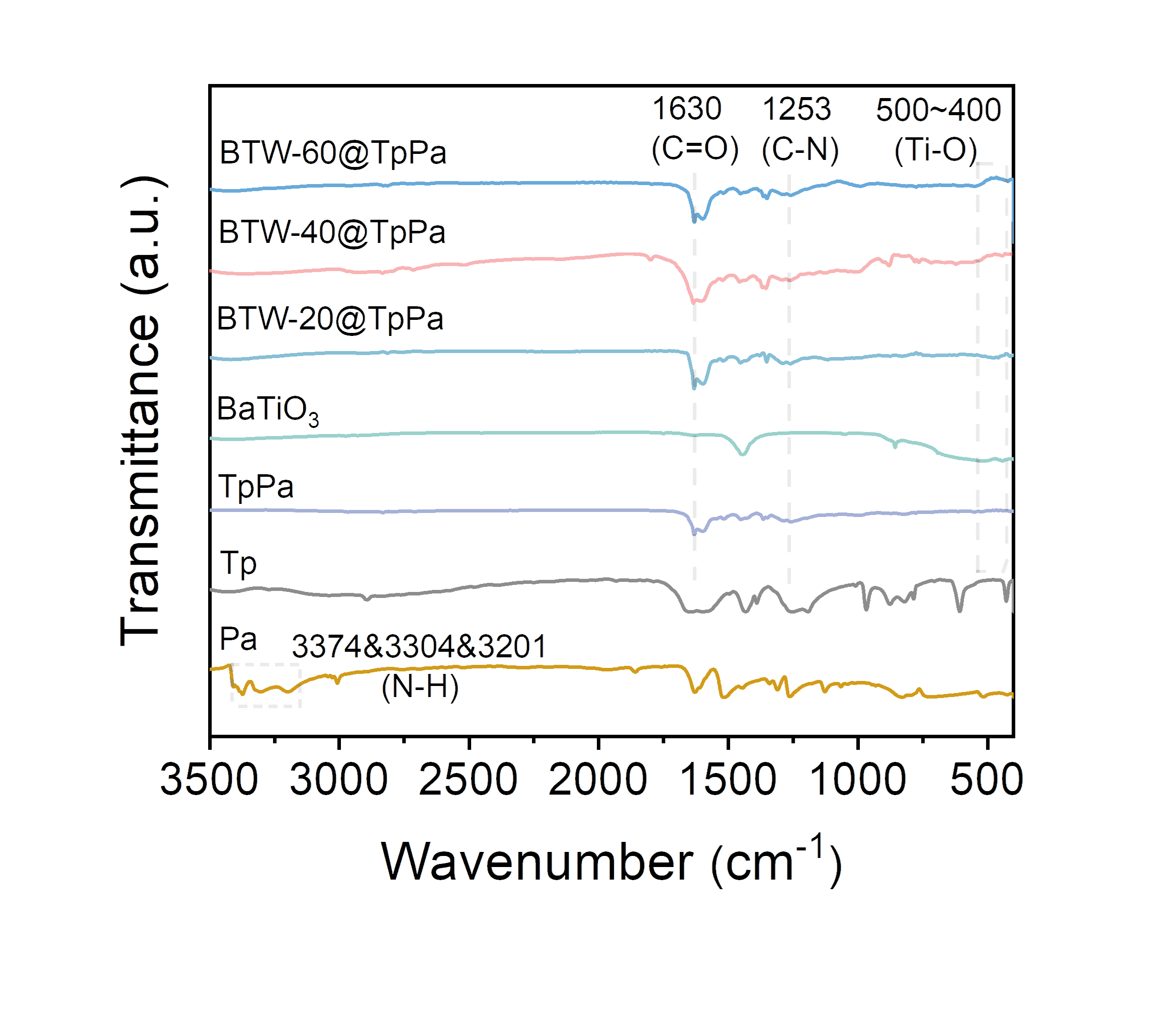


**Figure S9.** FT-IR spectra of BaTiO_3_, TpPa, and their heterostructures. Newly formed absorption bands at 1630, 1253, and 400 cm^-1^, assigned to C=O, C-N, and Ti-O, suggest the successful combination of BaTiO_3_ and TpPa.


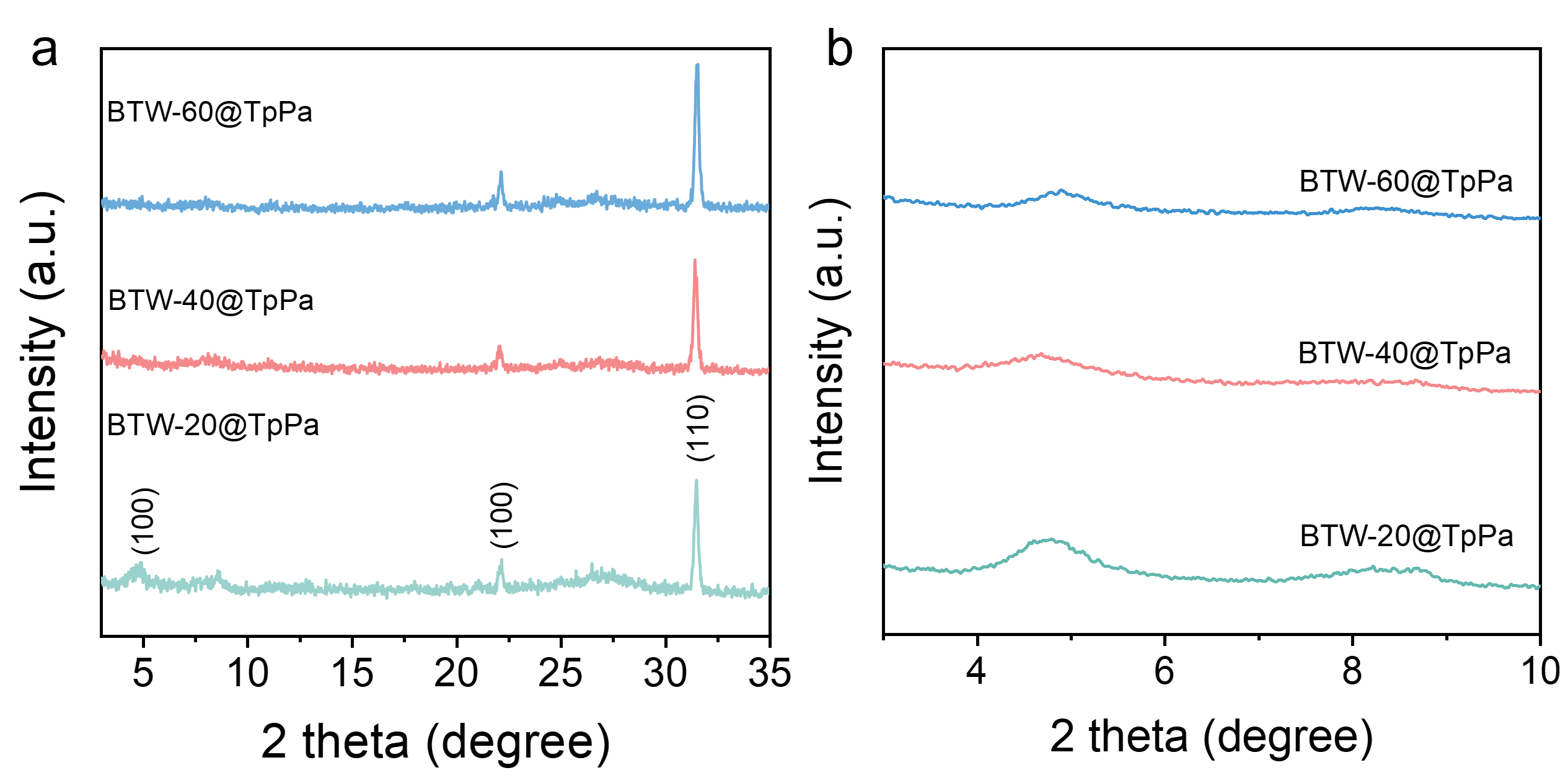


**Figure S10.** PXRD patterns of BTW-20@TpPa, BTW-40@TpPa, BTW-60@TpPa. (**a**) wide-angle region (3~35°). (**b**) small-angle region (3~10°). It should be noted that increasing the BaTiO_3_ content leads to a significant enhancement in the intensity of its characteristic diffraction peaks, while the intensity of the TpPa diffraction peak decreases substantially, eventually becoming undetectable.


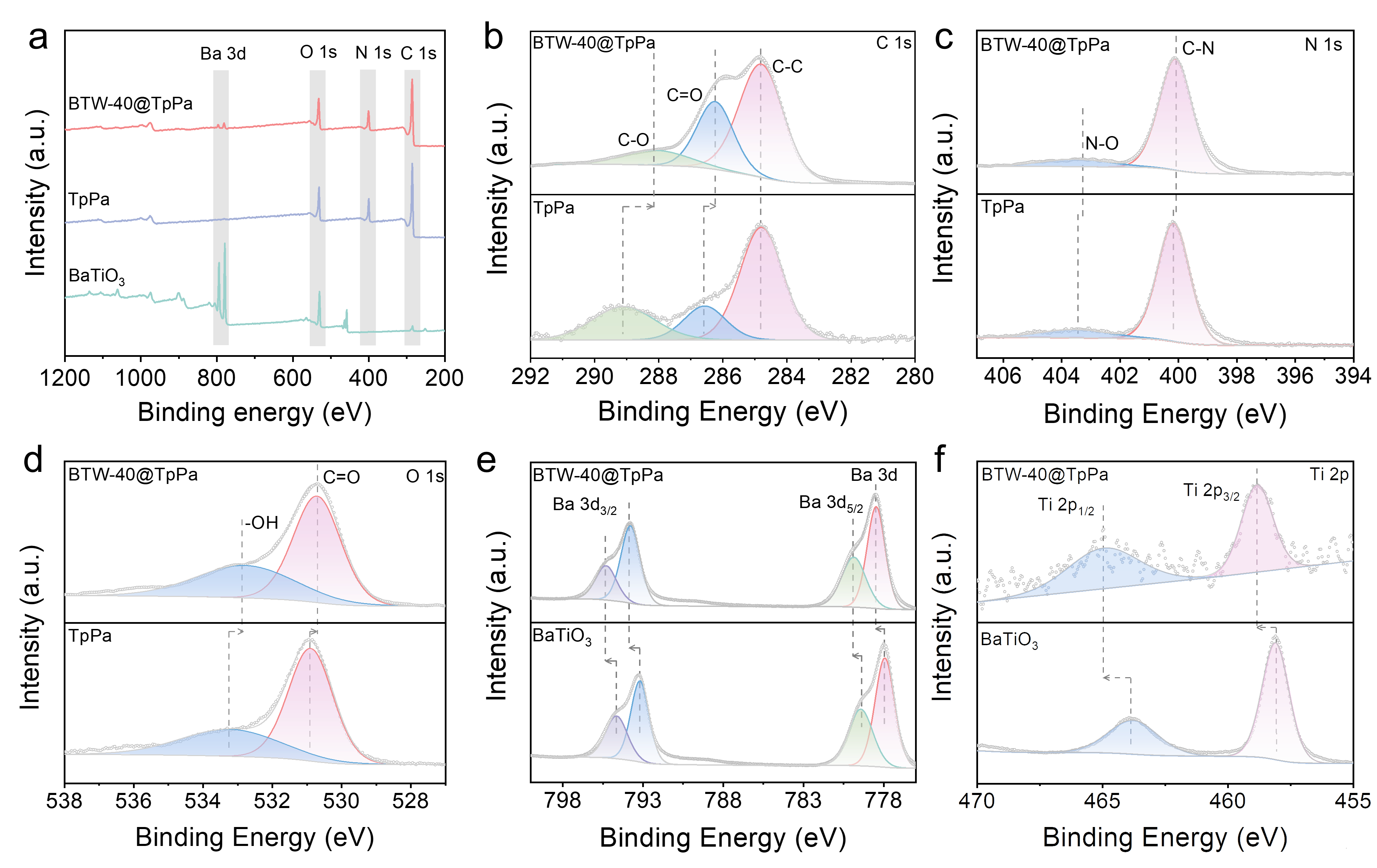


**Figure S11.** XPS spectra of BaTiO_3_, TpPa and BTW-40@TpPa. (**a**) Survey spectra, (**b**) C 1s, (**c**) N 1s, (**d**) O 1s, (**e**) Ba 3d, (**f**) Ti 2p. It is worth noting that the binding energies of C 1s, N 1s and O 1s in BTW-40@TpPa are negatively shifted compared with TpPa, while that of Ba 3d and Ti 2p are positively shifted compared with BaTiO_3_. This suggests that the electrons transfer from BaTiO_3_ to TpPa, further indicating the formation of heterostructures.


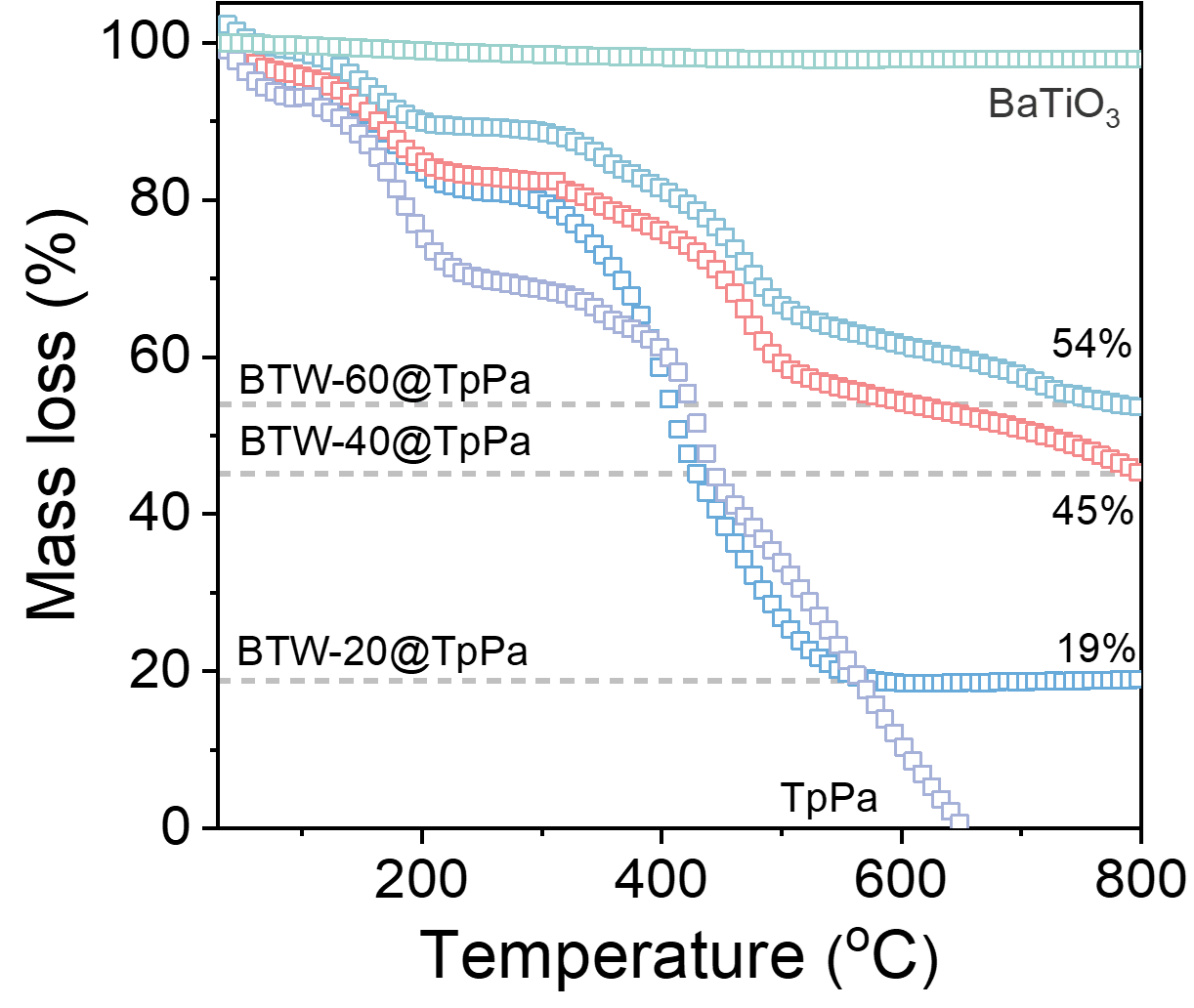


**Figure S12.** TGA curves of BaTiO_3_, TpPa and their heterostructures. The relative mass ratio between TpPa and BaTiO_3_ can be calculated from the difference of mass loss at 800°C. BaTiO_3_ has almost no mass loss during the entire heat-treatment process, whereas TpPa decomposes thoroughly when the temperature elevated above 700°C. It is evident that the BTW-40@TpPa losses of up to 55% at 800°C, indicating that approximately 45% of the BaTiO_3_ remains, that is close to the experimental theoretical value (40%).


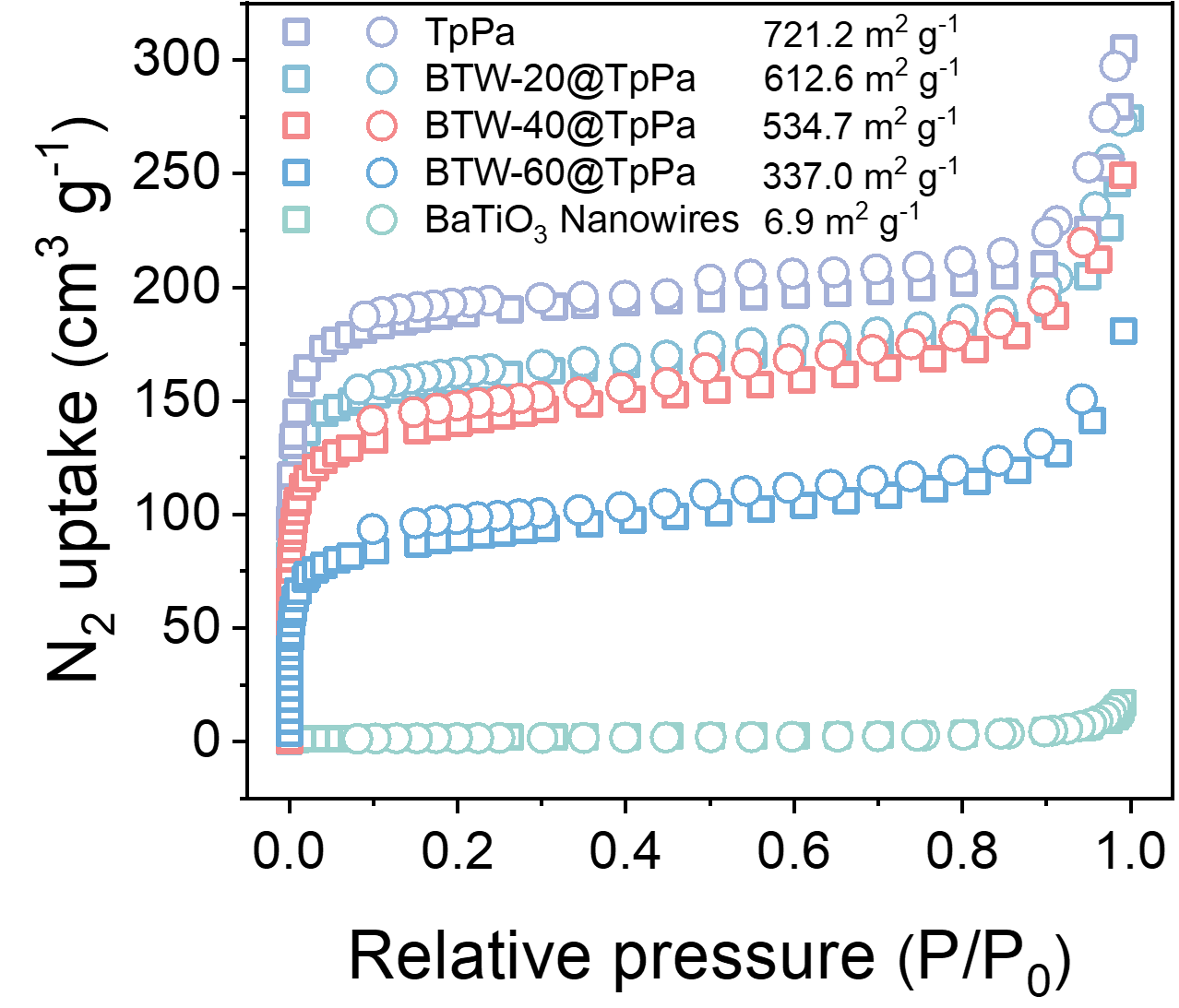


**Figure S13.** N_2_ adsorption and desorption curves recorded at 77 K of BaTiO_3_, TpPa, and their heterostructures.


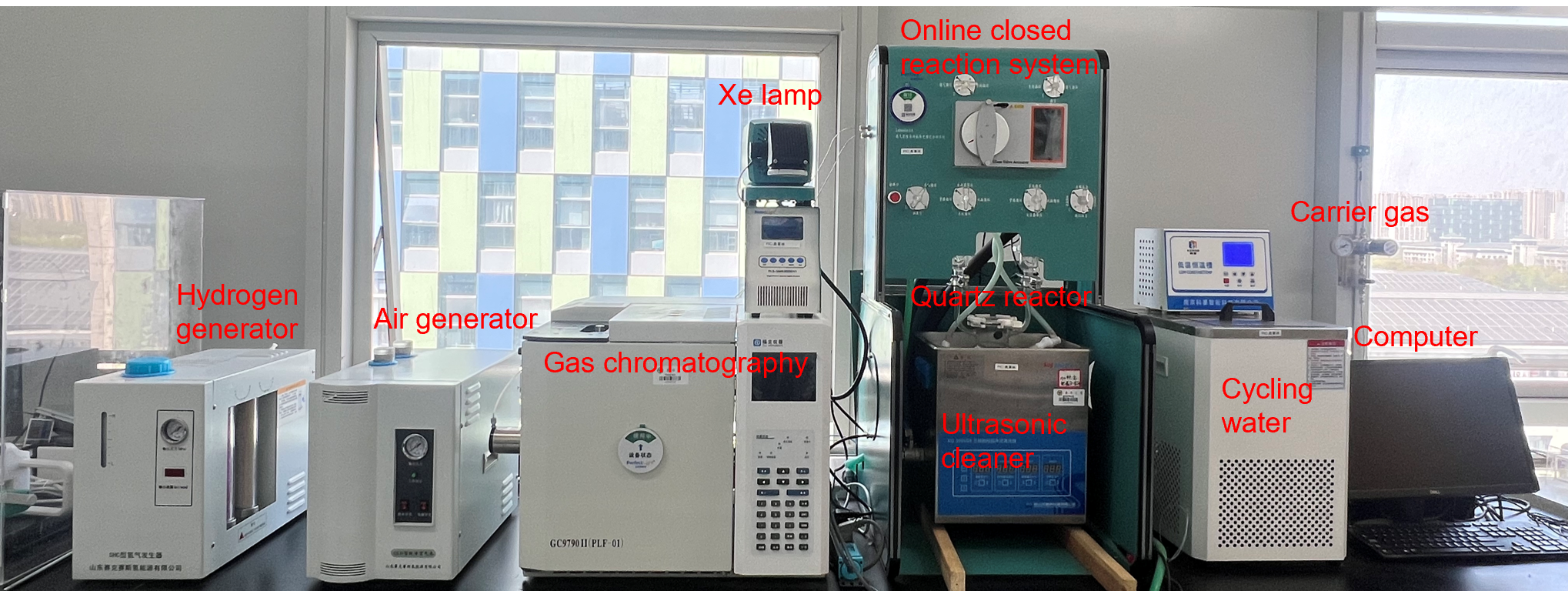


**Figure S14.** The apparatus of piezo-photocatalysis.


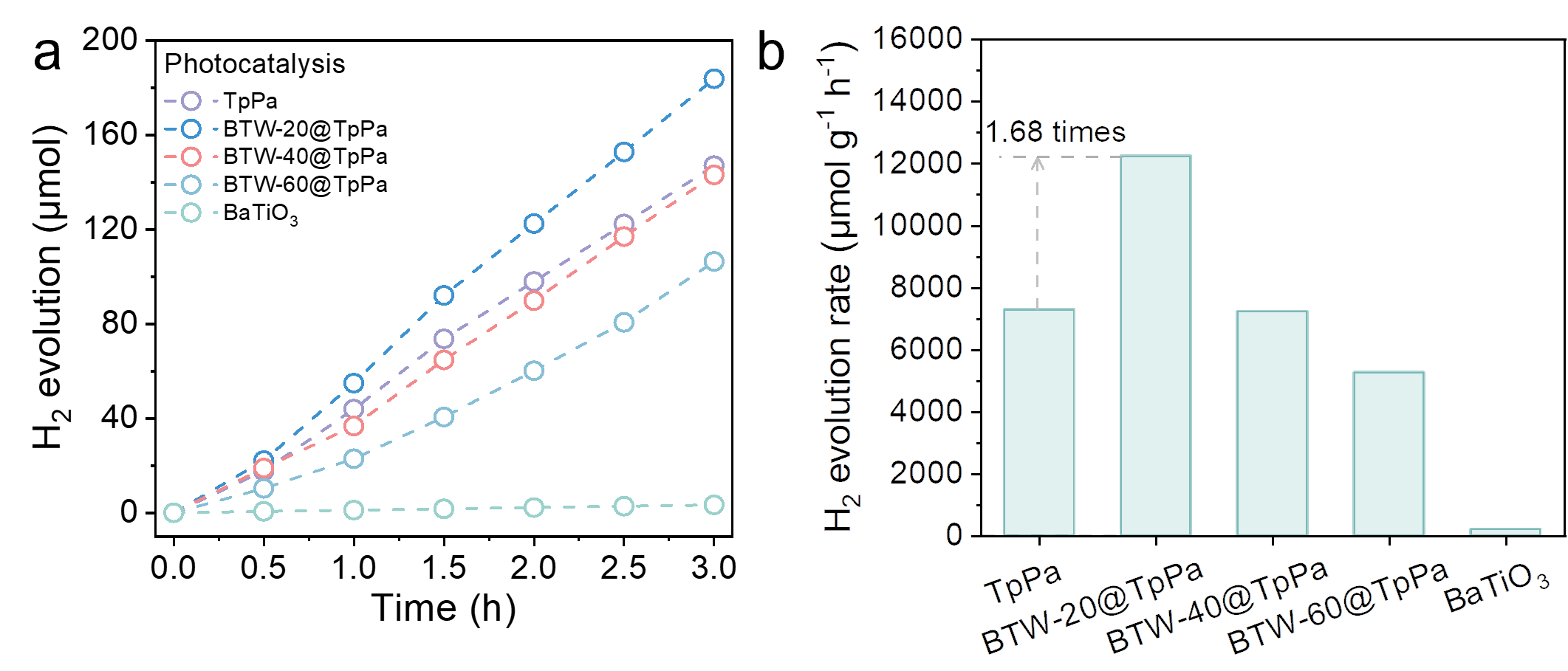


**Figure S15.** (**a**) Time-dependent photocatalytic H_2_ evolution and (**b**) the corresponding rates for BaTiO_3_, TpPa, BTW-20@TpPa, BTW-40@TpPa, and BTW-60@TpPa.


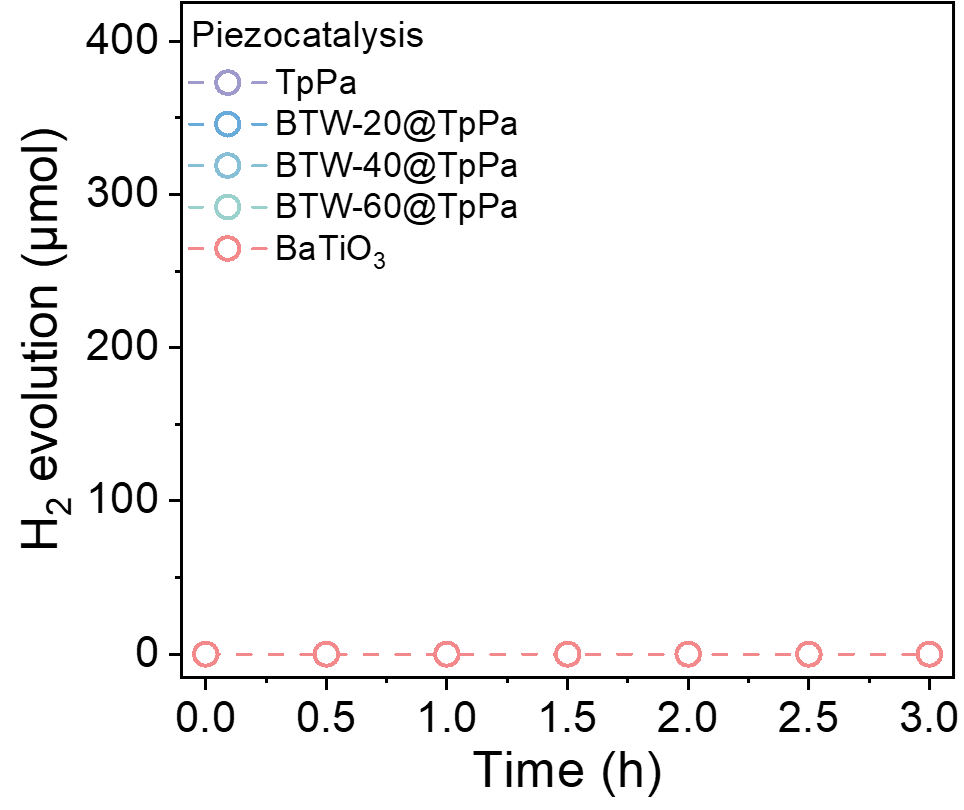


**Figure S16.** Time-dependent piezocatalytic H_2_ evolution for BaTiO_3_, TpPa, BTW-20@TpPa, BTW-40@TpPa, and BTW-60@TpPa.


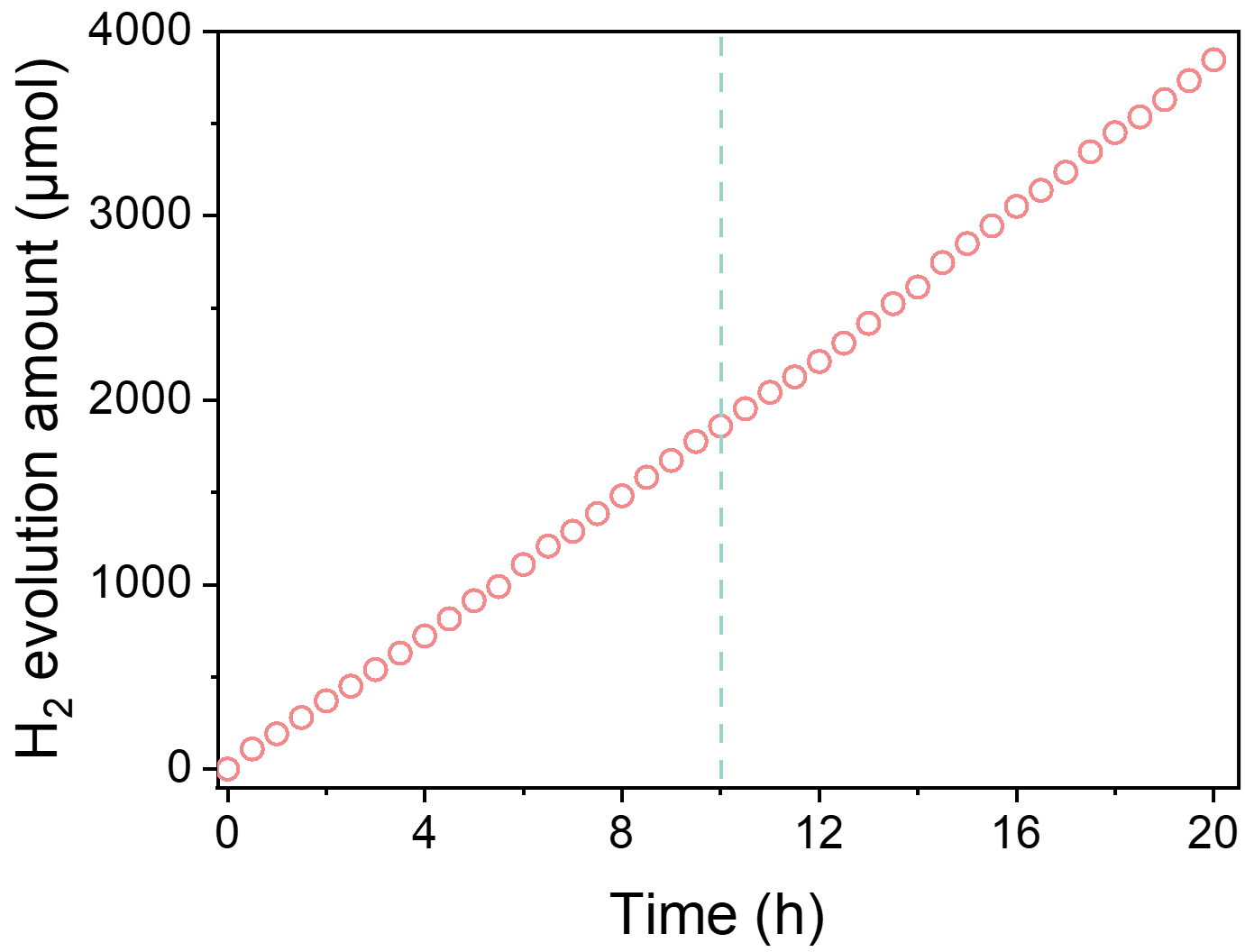


**Figure S17.** Long-time operation testing of piezo-photocatalytic H_2_ evolution. The green dashed vertical lines denote the supplement of AA sacrificial agent.


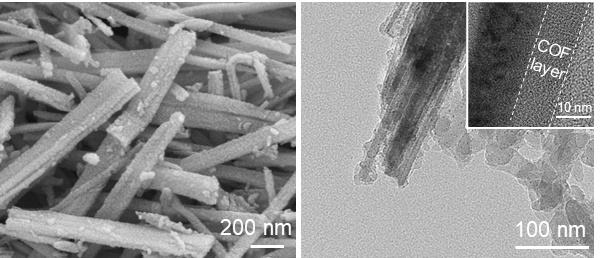


**Figure S18.** The SEM and TEM images of BTW-40@TpPa after 20 h piezo-photocatalytic reaction.


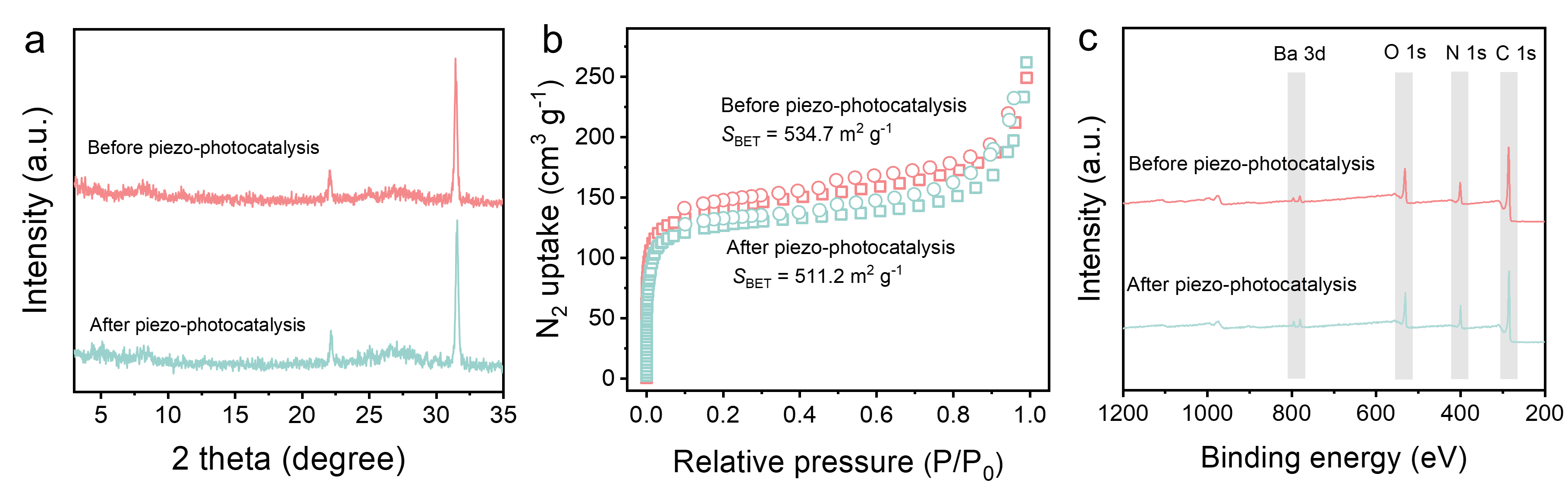


**Figure S19.** (**a**) XRD patterns and (**b**) N_2_ adsorption-desorption isotherms at 77 K, and (**c**) XPS spectra after long-time piezo-photocatalytic reaction.


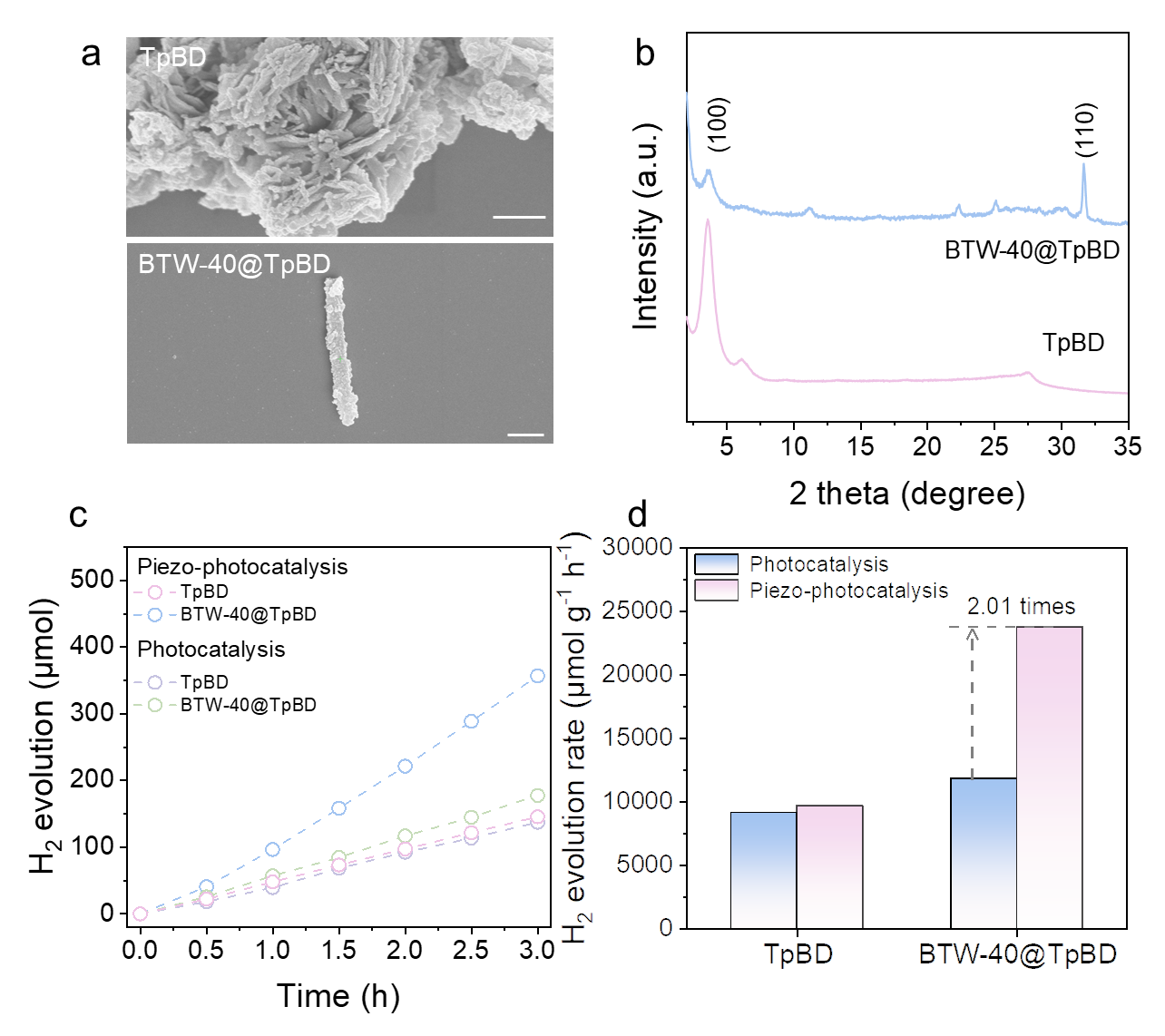


**Figure S20.** Piezo-photocatalytic performance of BTW-40@TpBD. (**a**) The morphologyle (scale bars are 500 nm) and (**b**) PXRD patterns of TpBD and BTW-40@TpBD. (**c**, **d**) Piezo-photocatalytic and photocatalytic activity of TpBD and BTW-40@TpBD.


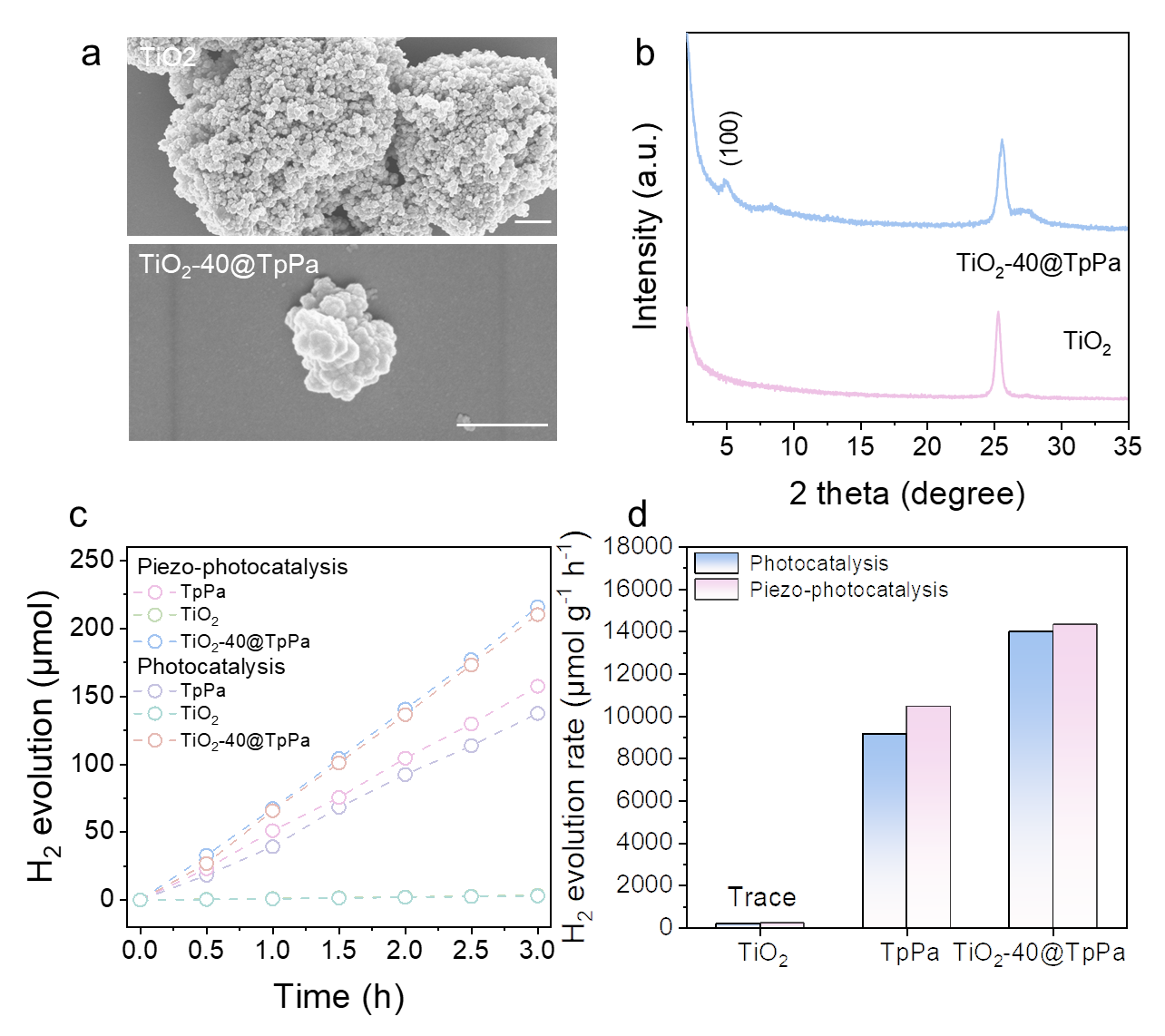


**Figure S21.** Piezo-photocatalytic performance of TiO_2_-40@TpPa. (a) The morphology (scale bars are 500 nm) and (b) PXRD patterns of TpPa and TiO_2_-40@TpPa. (c, d) Piezo-photocatalytic and photocatalytic activity of TpPa and TiO_2_-40@TpPa.


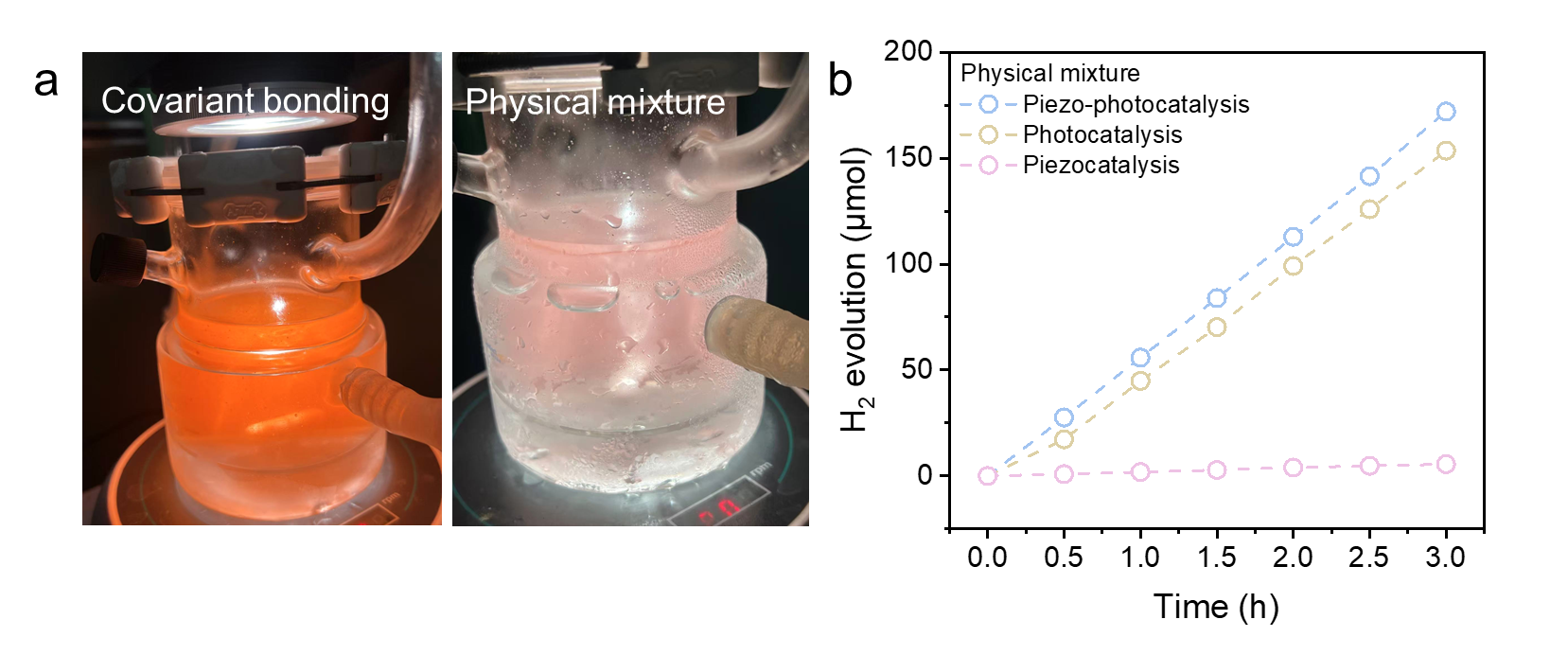


**Figure S22.** (**a**) The color comparison of physically mixed and covalently bonded prepared catalysts in catalytic systems. (**b**) The H_2_ evolution performance of physical mixture under photocatalytic, piezocatalytic and piezo-photocatalytic conditions.


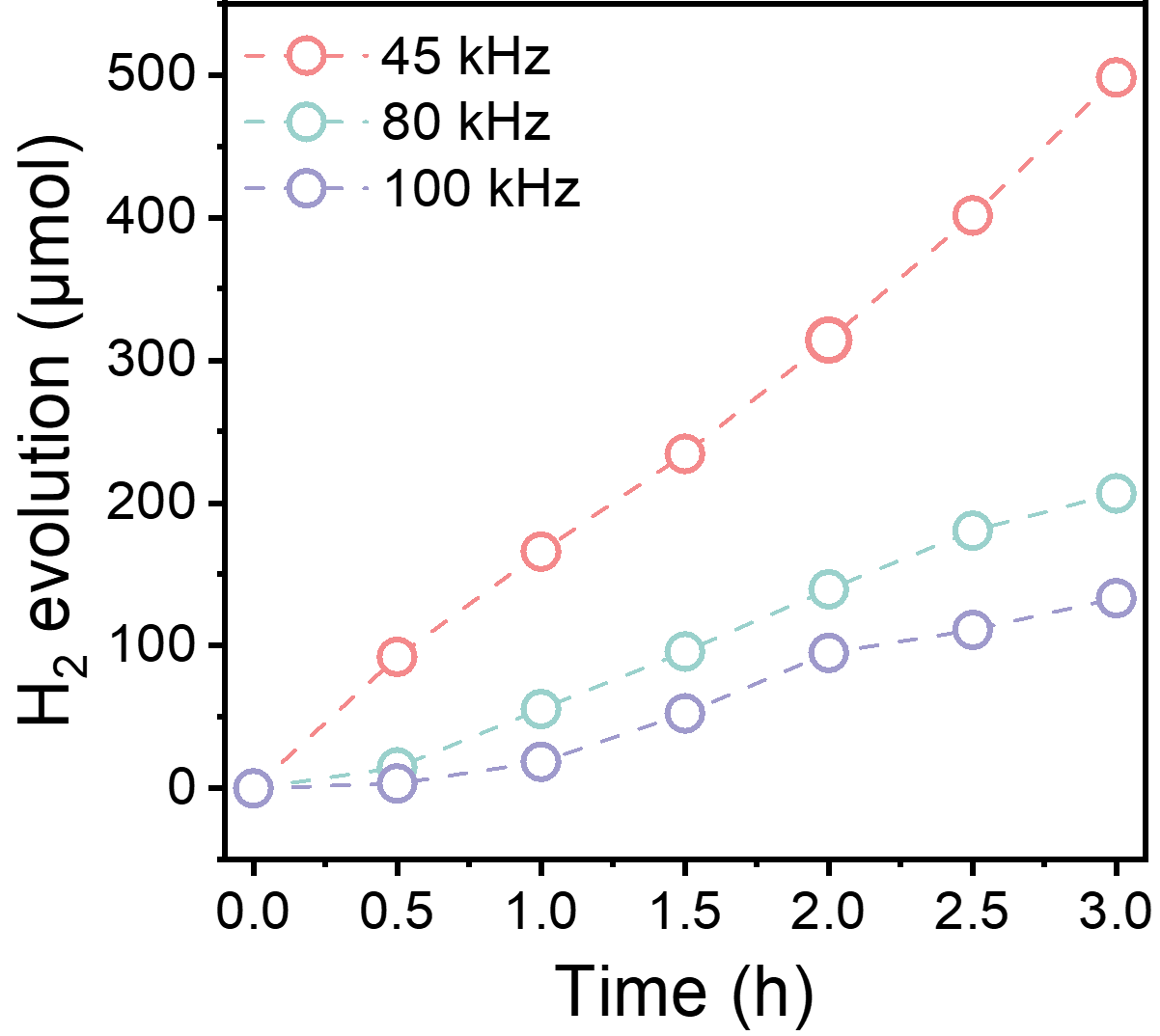


**Figure S23.** The piezo-photocatalytic H_2_ evolution of BTW-40@TpPa under different ultrasonic frequency.


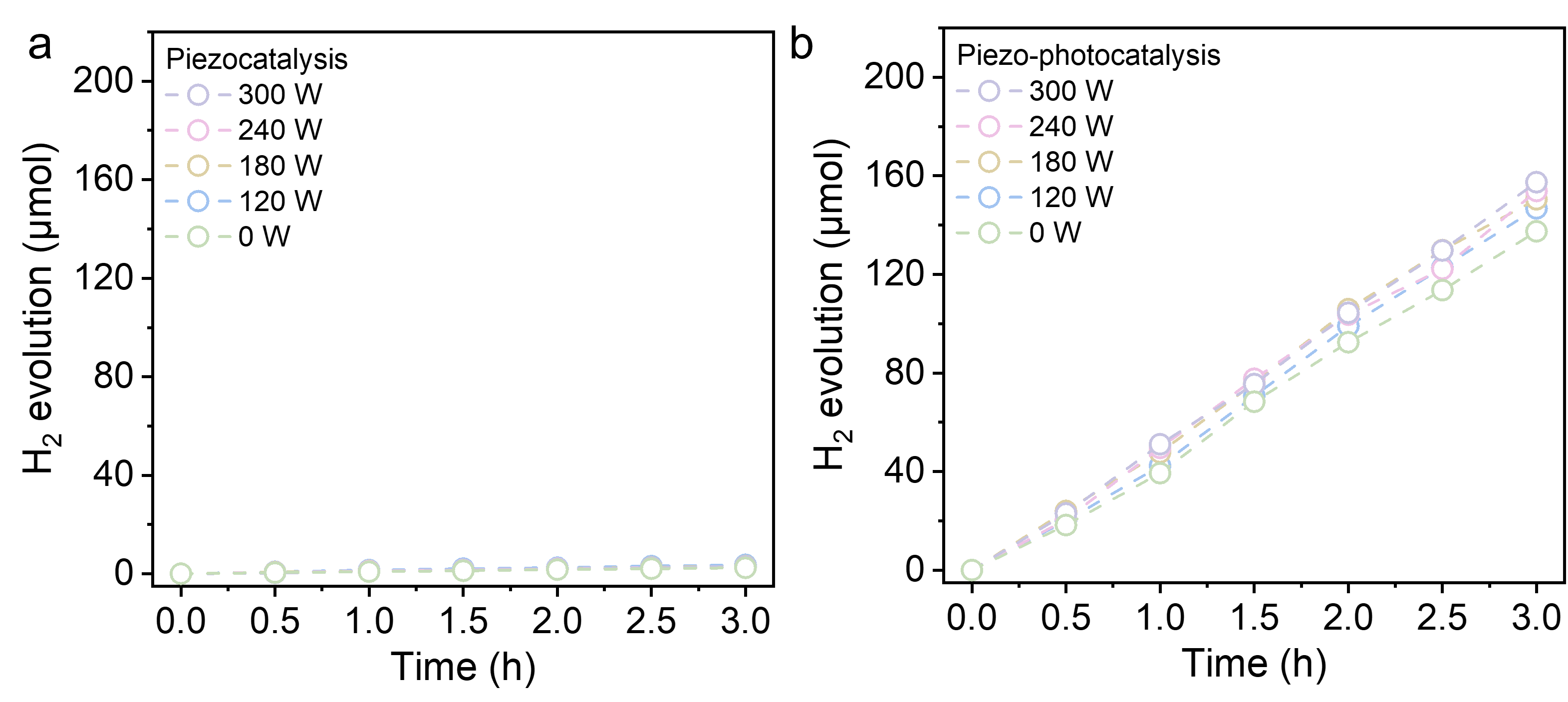


**Figure S24.** Catalytic performance of TpPa under different ultrasonic power. Time-dependent (**a**) piezocatalytic and (**b**) piezo-photocatalytic H_2_ evolution.


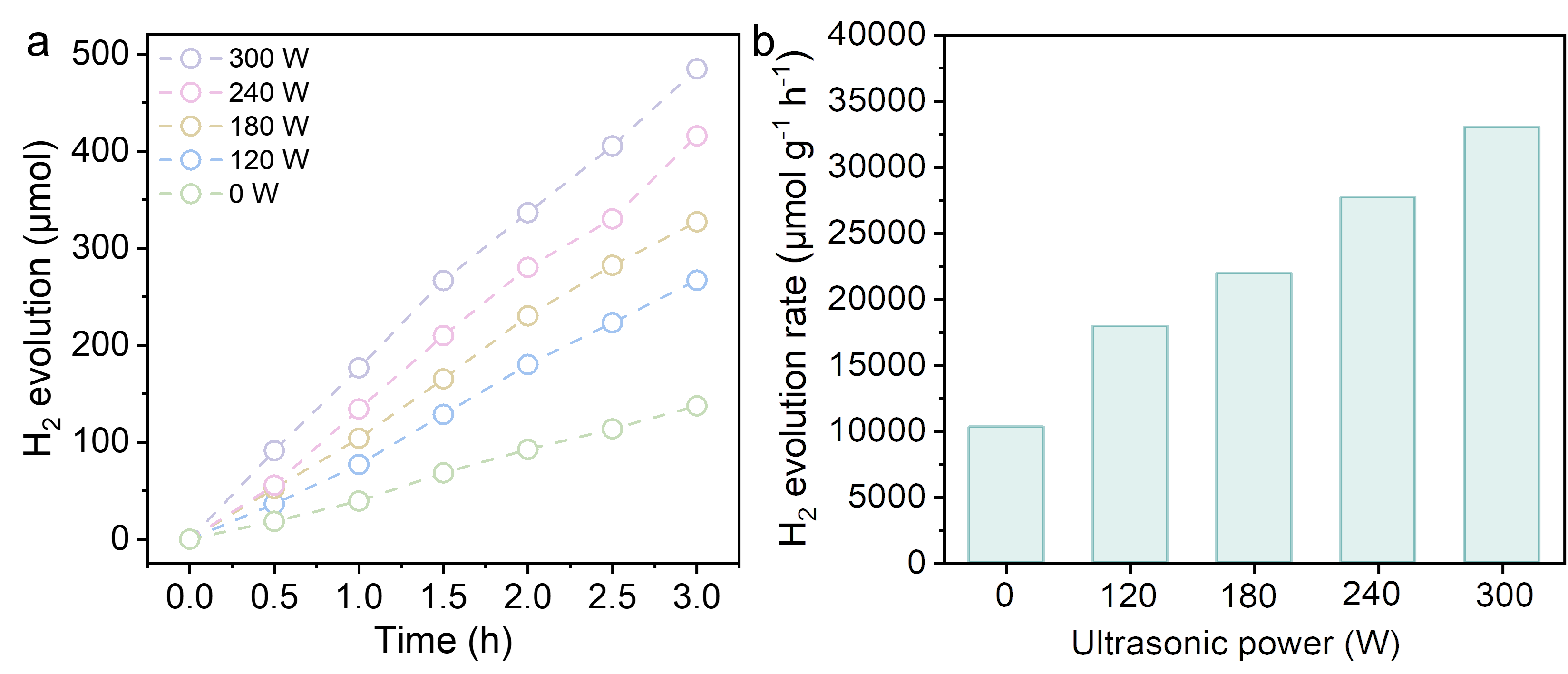


**Figure S25.** Piezo-photocatalytic performance of BTW-40@TpPa under different ultrasonic power. (**a**) Time-dependent piezo-photocatalytic H_2_ evolution. (**b**) The corresponding mass-normalized rate.


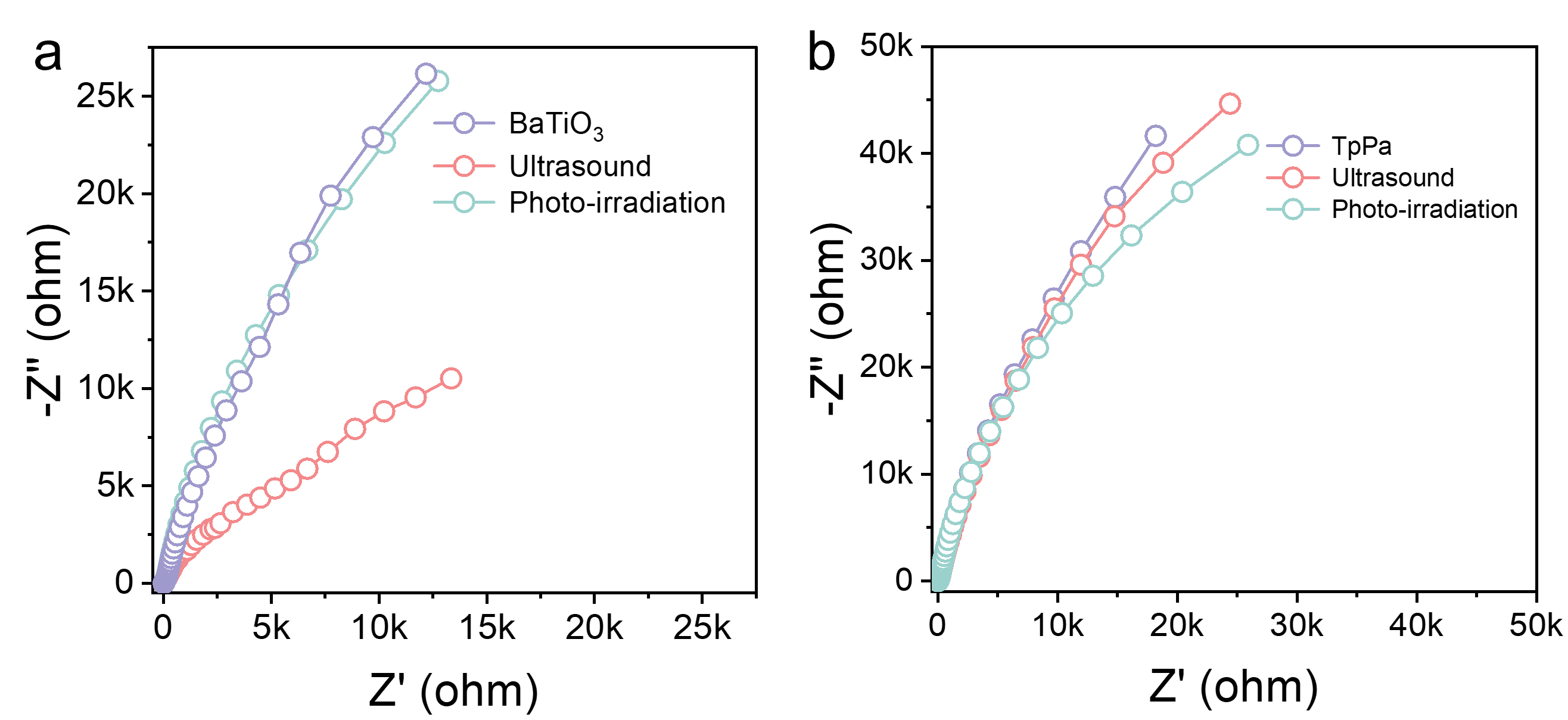


**Figure S26.** The EIS analysis of (**a**) BaTiO_3_ and (**b**) TpPa under different operation conditions.


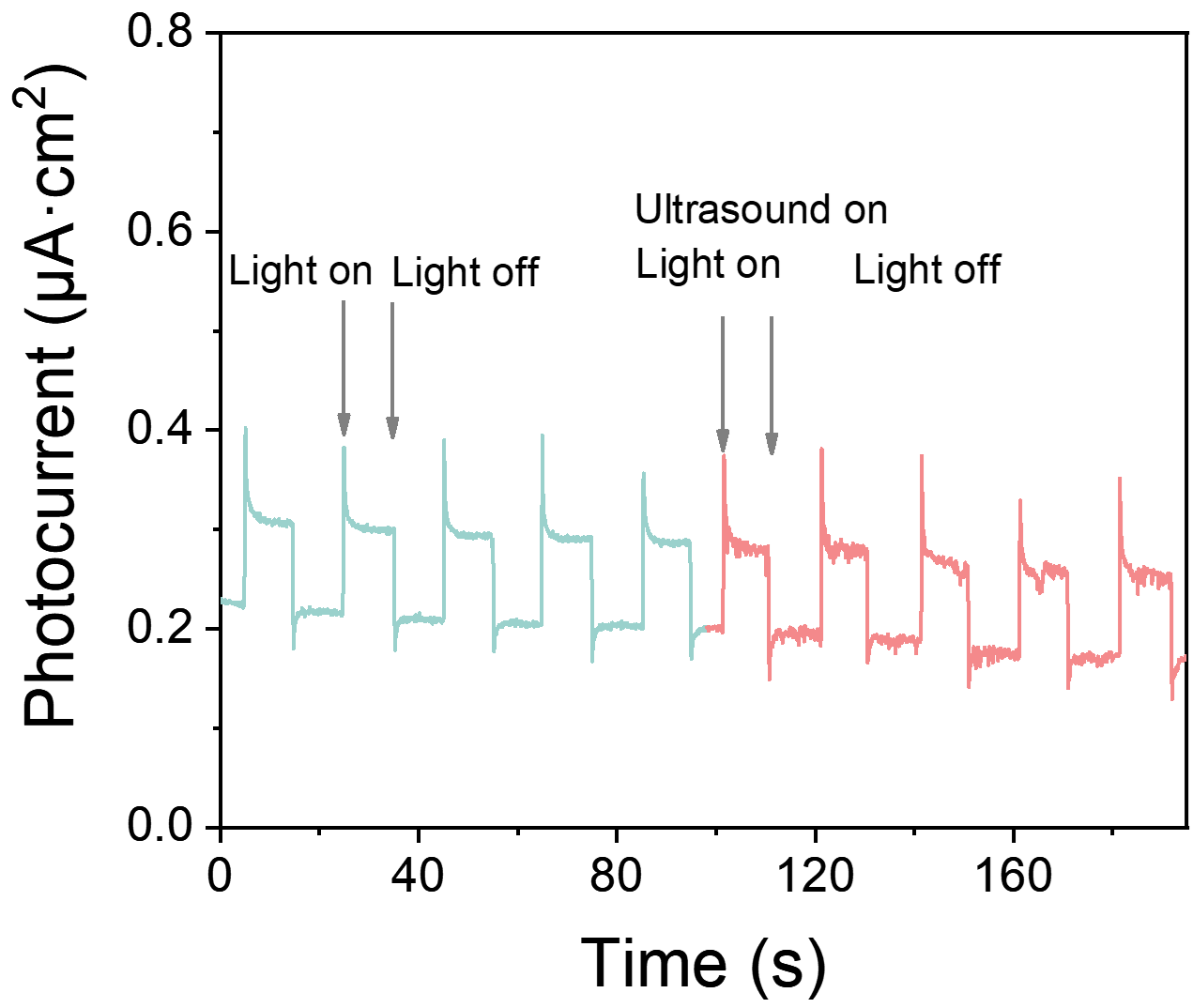


**Figure S27.** The photocurrent and piezo-photo-current analysis of TpPa.


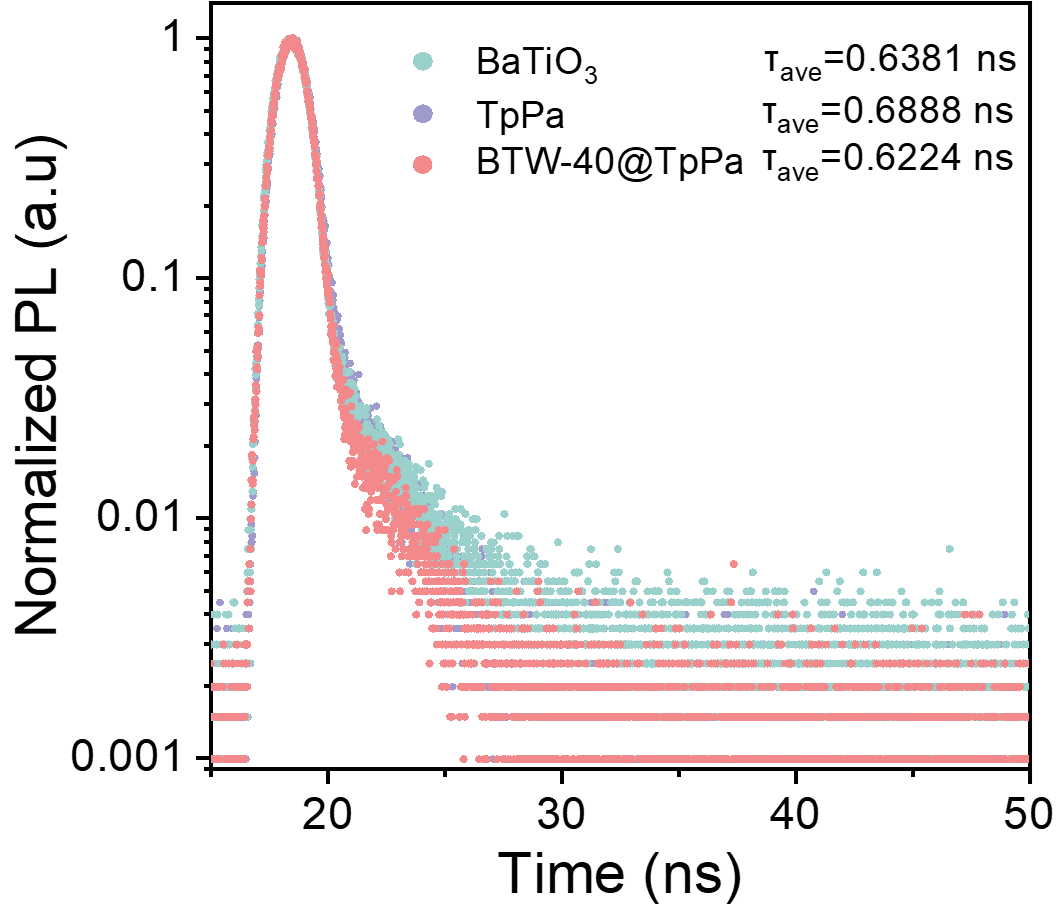


**Figure S28.** Time-resolved PL decay spectra of BaTiO_3_, TpPa, and BTW-40@TpPa.


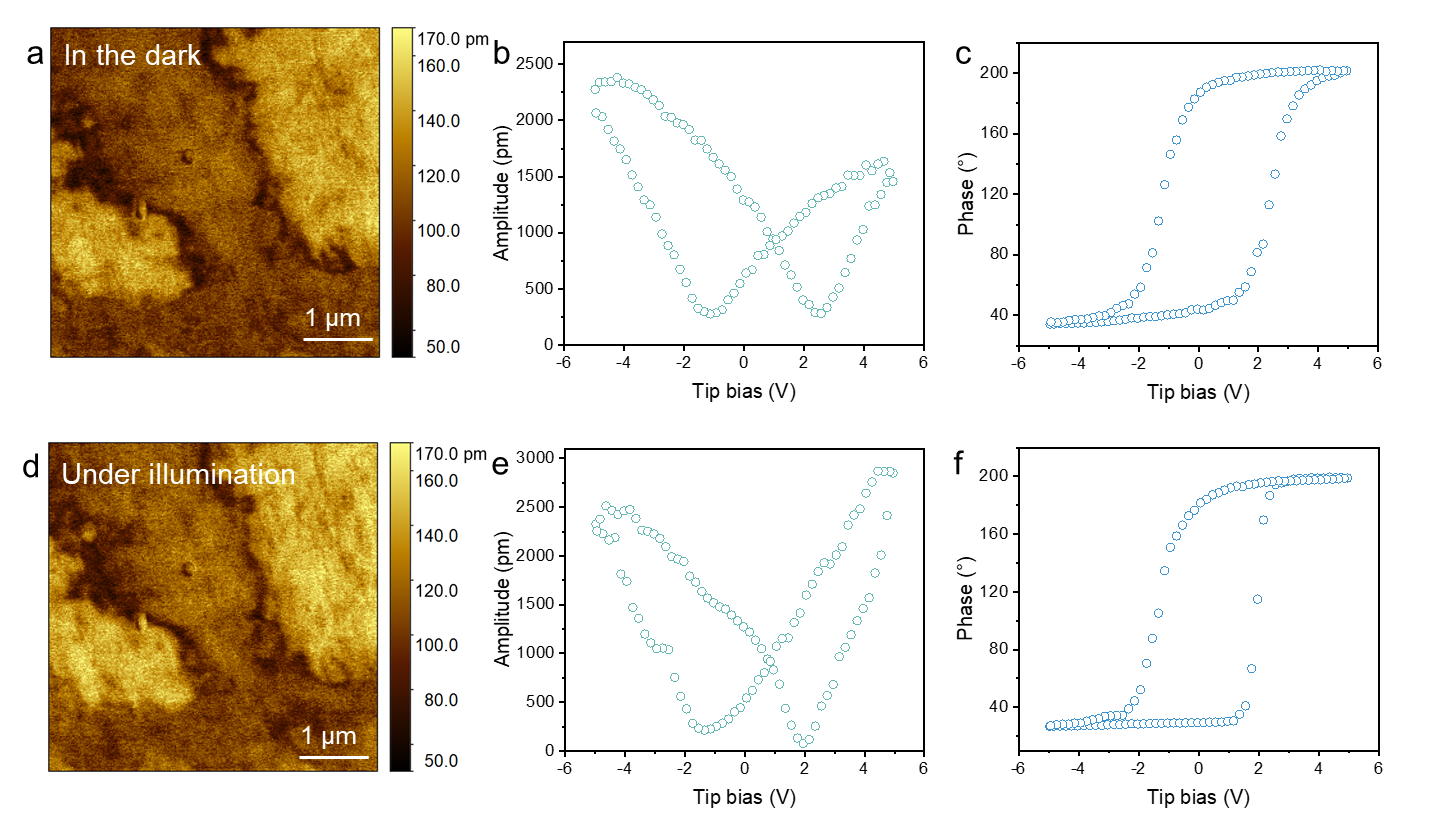


**Figure S29.** (**a**, **d**) The PFM images, (**b**, **e**) amplitude-voltage and (**c**, **f**) phase curve in the dark and under illumination.


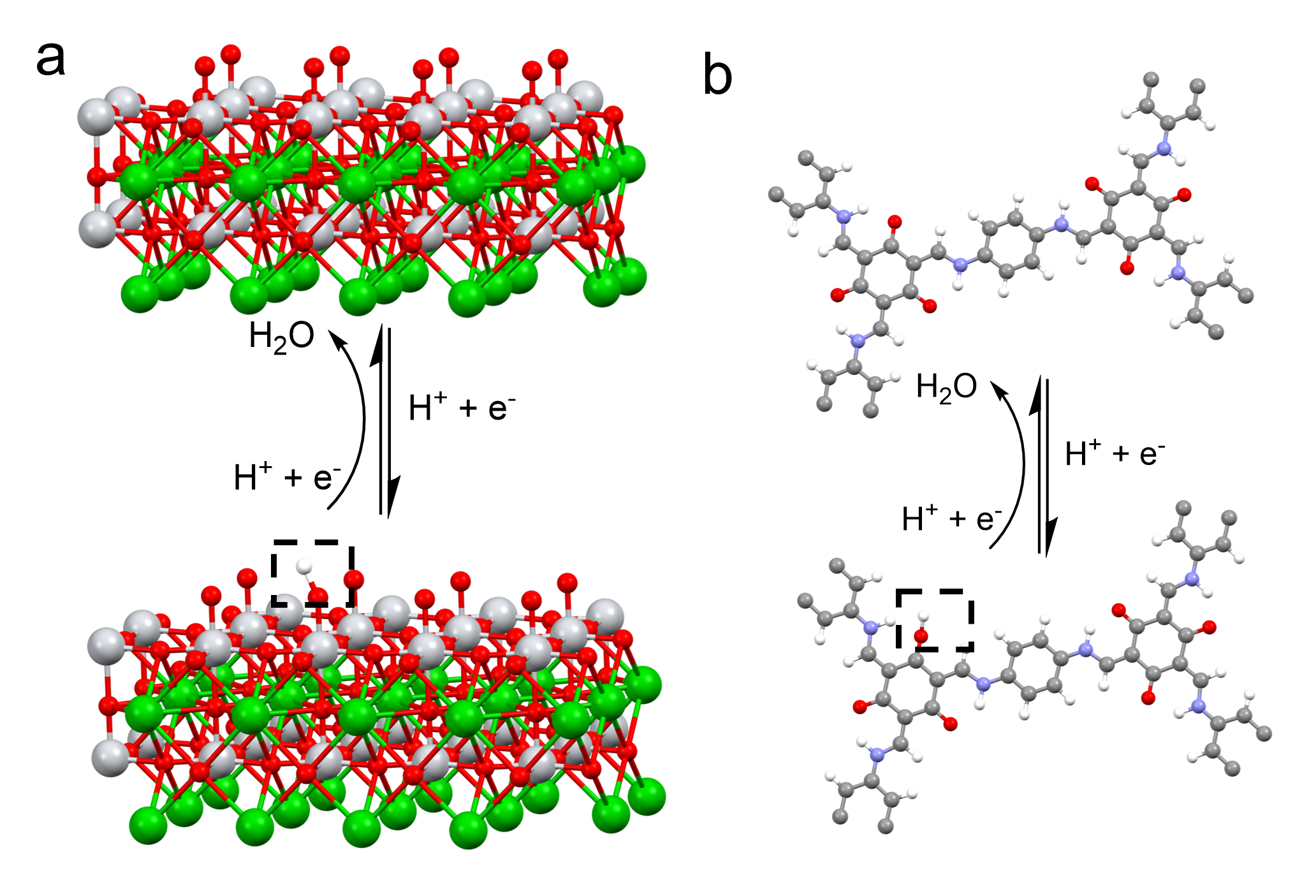


**Figure S30.** Proposed photocatalytic pathways of HER with the most energetically favorable absorbed intermediates (*H) in (**a**) BaTiO_3_ and (**b**) TpPa.

**
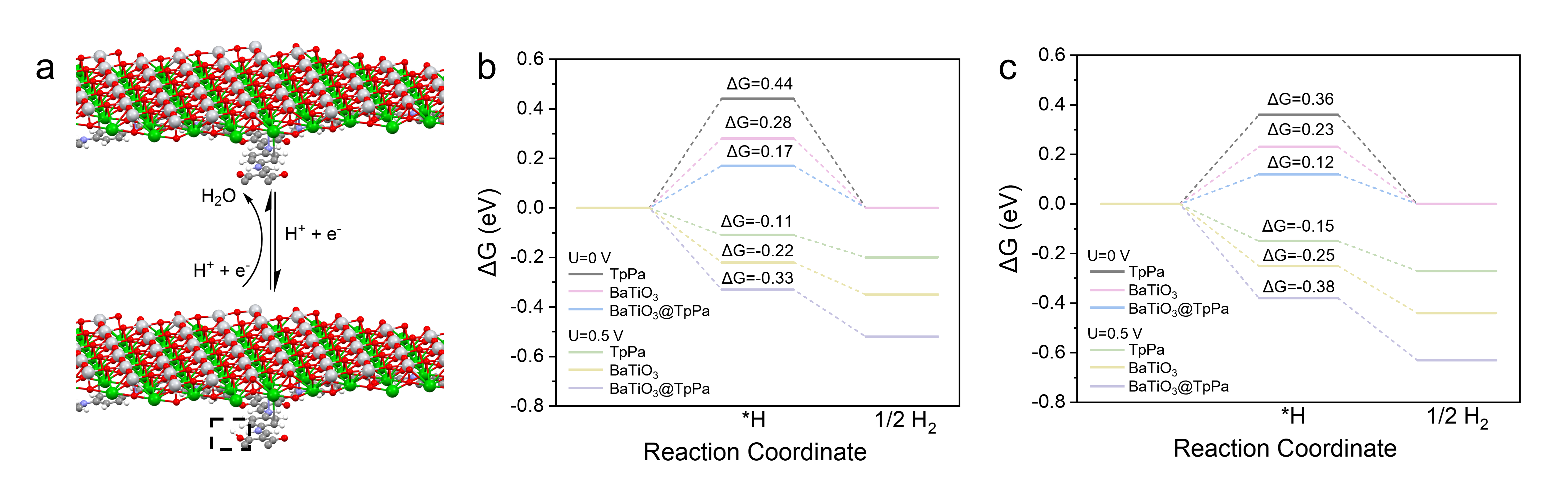
**

**Figure S31.** (**a**) Proposed photocatalytic pathways of HER process with the most energetically favorable absorbed intermediates in BaTiO_3_@TpPa. The calculated Gibbs free energy changes of intermediate states involved in HER processes (**b**) without and (**c**) with piezoelectric effect for BaTiO_3_, TpPa and BaTiO_3_@TpPa.

1. **Supporting Tables**

**Table S1** Calculated AQY of BTW-40@TpPa sample at different wavelengths when only light illumination.

| Wavelength (nm) | Light intensity (W m^-2^) | H_2_ evolution  (μmol s^-1^) | AQY  (%) |
| --- | --- | --- | --- |
| 420 | 299.36 | 0.0138 | 0.933 |
| 500 | 343.95 | 0.0121 | 0.597 |
| 550 | 382.16 | 0.00683 | 0.276 |
| 600 | 321.66 | 0.00119 | 0.052 |

**Table S2** Calculated AQY of BTW-40@TpPa sample at different wavelengths when synergistic ultrasound and light illumination.

| Wavelength (nm) | Light intensity (W m^-2^) | H_2_ evolution  (μmol s^-1^) | AQY  (%) |
| --- | --- | --- | --- |
| 420 | 299.36 | 0.139 | 9.387 |
| 500 | 343.95 | 0.105 | 5.163 |
| 550 | 382.16 | 0.040 | 1.619 |
| 600 | 321.66 | 0.011 | 0.479 |

**Table S3** Comparisons of H_2_ evolution performance with previously reported piezo-photocatalysts

| Sample | Light | Sacrificial agents | Co-catalyst | H_2_ evolution rate  (μmol·g^-1^·h^-1^) | Refs |
| --- | --- | --- | --- | --- | --- |
| BTW-40@TpPa | λ > 420 nm | Ascorbic acid | 0.5 wt% Pt | 32997 | **This work** |
| TPCNNS | λ > 420 nm | Sodium ascorbate | 3 wt% Pt | 1153 | [5] |
| MoS_2_-3%/TpPa | λ > 420 nm | Ascorbic acid | 3 wt% Pt | 5580 | [6] |
| 20% NCM/TpPa | λ > 420 nm | Ascorbic acid | Pt | 15300 | [7] |
| Ti-MOF/TpPa | λ > 400 nm | Sodium ascorbate | 1.2 wt% | 13980 | [8] |
| TiO_2_-TpPa-1-COF (1:3) | λ > 420 nm | Sodium ascorbate | 3 wt% | 11190 | [9] |
| MOF-808@TpPa | λ > 420 nm | Sodium ascorbate | 1 wt% | 11880 | [10] |
| TpPa-Cu(II)-COF | λ > 420 nm | L-/D-cysteine | 10.76 wt% Cu | 14720 | [11] |
| g-C_40_N_3_-COF | λ > 420 nm | Triethanolamine | 3 wt% Pt | 4120 | [12] |
| P-COF-1/CTF | λ > 420 nm | Triethanolamine | Pt | 14100 | [13] |
| LN-type ZnSnO_3_ NWs | λ > 420 nm | N.A. | N.A. | 3453 | [14] |
| UT-g-C_3_N_4_ | λ > 420 nm | Glucose | N.A. | 12160 | [15] |
| TiO_2_@MoSe_2_ | λ > 420 nm | Methanol | N.A. | 2196 | [16] |
| GD-C_3_N_4_ | λ > 420 nm | Triethanolamine | 3 wt% | 23060 | [17] |

Note: N.A.: not available.

**Table S4** The fitting data of electrochemical impedance spectroscopy of BTW-40@TpPa.

| Conditions | R_S_  (Ω·cm^2^) | CPEf  (Ω^-1^ cm^-2^ s^-n^) | R_f_  (Ω cm^2^) | CPEdl  (Ω^-1^ cm^-2^ s^-n^) | R_ct_  (Ω cm^2^) |
| --- | --- | --- | --- | --- | --- |
| BTW-40@TpPa | 14.93 | 9.336×10^-5^ | 5.09 | 5.033×10^-4^ | 53795 |
| Ultrasound | 15.03 | 1.707×10^-4^ | 6.60 | 1.079×10^-3^ | 49953 |
| Photo-irradiation | 14.51 | 8.146×10^-5^ | 7.017 | 8.130×10^-4^ | 43279 |
| Ultrasound and photo-irradiation | 14.18 | 6.854×10^-5^ | 7.41 | 8.697×10^-4^ | 37811 |

1. **References**

[1] a) N. Bao, L. Shen, G. Srinivasan, K. Yanagisawa, A. Gupta, *J. Phys. Chem. C* **2008**, *112* (23), 8634; b) H. Tang, Y. Lin, H. A. Sodano, *Adv. Energy Mater.* **2013**, *3* (4), 451.

[2] S. Kandambeth, A. Mallick, B. Lukose, M. V. Mane, T. Heine, R. Banerjee, *J. Am. Chem. Soc.* **2012**, *134* (48), 19524.

[3] Y. Wang, X. Li, Y. Chen, Y. Li, Z. Liu, C. Fang, T. Wu, H. Niu, Y. Li, W. Sun, W. Tang, W. Xia, K. Song, H. Liu, W. Zhou, *Adv. Mater.* **2023**, *35* (45), 2305257.

[4] Y. Wang, Y. Xu, S. Dong, P. Wang, W. Chen, Z. Lu, D. Ye, B. Pan, D. Wu, C. D. Vecitis, G. Gao, *Nat. Commun.* **2021**, *12* (1), 3508.

[5] P. Dong, A. Zhang, T. Cheng, J. Pan, J. Song, L. Zhang, R. Guan, X. Xi, J. Zhang, *Chin. J. Catal.*  *43* (10), 2592.

[6] M.-Y. Gao, C.-C. Li, H.-L. Tang, X.-J. Sun, H. Dong, F.-M. Zhang, *J. Mater. Chem. A* **2019**, *7* (35), 20193.

[7] H. Hu, X. Zhang, K. Zhang, Y. Ma, H. Wang, H. Li, H. Huang, X. Sun, T. Ma, *Adv. Energy. Mater.* **2024**, *14* (11), 2303638.

[8] C.-X. Chen, Y.-Y. Xiong, X. Zhong, P. C. Lan, Z.-W. Wei, H. Pan, P.-Y. Su, Y. Song, Y.-F. Chen, A. Nafady, Sirajuddin, S. Ma, *Angew. Chem. Int. Ed.* **2022**, *61* (3), e202114071.

[9] C.-C. Li, M.-Y. Gao, X.-J. Sun, H.-L. Tang, H. Dong, F.-M. Zhang, *Appl. Catal. B Environ.* **2020**, *266*, 118586.

[10] H.-Y. Zhang, Y. Yang, C.-C. Li, H.-L. Tang, F.-M. Zhang, G.-L. Zhang, H. Yan, *J. Mater. Chem. A* **2021**, *9* (31), 16743.

[11] W. Weng, J. Guo, *Nat. Commun.* **2022**, *13* (1), 5768.

[12] S. Bi, C. Yang, W. Zhang, J. Xu, L. Liu, D. Wu, X. Wang, Y. Han, Q. Liang, F. Zhang, *Nat. Commun.* **2019**, *10* (1), 2467.

[13] H. Li, H. Tian, X. Wang, M. Pi, S. Wei, H. Zhu, D. Zhang, S. Chen, *ACS Appl. Energy. Mater.* **2019**, *2* (7), 4692.

[14] Y.-C. Wang, J. M. Wu, *Adv. Funct. Mater.* **2020**, *30* (5), 1907619.

[15] C. Hu, F. Chen, Y. Wang, N. Tian, T. Ma, Y. Zhang, H. Huang, *Adv. Mater.* **2021**, *33* (24), 2101751.

[16] S.-L. Guo, S.-N. Lai, J. M. Wu, *ACS Nano* **2021**, *15* (10), 16106.

[17] Y. Yu, W. Yan, X. Wang, P. Li, W. Gao, H. Zou, S. Wu, K. Ding, *Adv. Mater.* **2018**, *30* (9), 1705060.
